# hCINAP regulates the DNA-damage response and mediates the resistance of acute myelocytic leukemia cells to therapy

**DOI:** 10.1038/s41467-019-11795-5

**Published:** 2019-08-23

**Authors:** Ruidan Xu, Shuyu Yu, Dan Zhu, Xinping Huang, Yuqi Xu, Yimin Lao, Yonglu Tian, Jinfang Zhang, Zefang Tang, Zemin Zhang, Jing Yi, Hong-Hu Zhu, Xiaofeng Zheng

**Affiliations:** 1 State Key Laboratory of Protein and Plant Gene Research, School of Life Sciences, Peking University, Beijing, 100871 China; 2 Department of Biochemistry and Molecular Biology, School of Life Sciences, Peking University, Beijing, 100871 China; 3 Shanghai Key Laboratory of Tumor Microenvironment and Inflammation, Department of Biochemistry and Molecular Cell Biology, Institutes of Medical Sciences, Shanghai Jiao Tong University School of Medicine, Shanghai, 200025 China; 4 Peking-Tsinghua Center for Life Sciences, Academy for Advanced Interdisciplinary Studies, Peking University, Beijing, 100871 China; 5 State Key Laboratory of Membrane Biology, PKU-IDG/McGovern Institute for Brain Research, School of Life Sciences, Peking University, Beijing, 100871 China; 6 School of Life Sciences and BIOPIC, Peking University, Beijing, 100871 China; 7 Peking University People’s Hospital, Peking University, Beijing, 100014 China

**Keywords:** Acute myeloid leukaemia, DNA damage response, Sumoylation

## Abstract

Acute myeloid leukemia (AML) is a genetically heterogeneous malignant disorder of the hematopoietic system, characterized by the accumulation of DNA-damaged immature myeloid precursors. Here, we find that hCINAP is involved in the repair of double-stranded DNA breaks (DSB) and that its expression correlates with AML prognosis. Following DSB, hCINAP is recruited to damage sites where it promotes SENP3-dependent deSUMOylation of NPM1. This in turn results in the dissociation of RAP80 from the damage site and CTIP-dependent DNA resection and homologous recombination. NPM1 SUMOylation is required for recruitment of DNA repair proteins at the early stage of DNA-damage response (DDR), and SUMOylated NPM1 impacts the assembly of the BRCA1 complex. Knockdown of hCINAP also sensitizes a patient-derived xenograft (PDX) mouse model to chemotherapy. In clinical AML samples, low hCINAP expression is associated with a higher overall survival rate in patients. These results provide mechanistic insight into the function of hCINAP during the DNA-damage response and its role in AML resistance to therapy.

## Abstract (web summary)

Acute myeloid leukemia cells are often resistant to radiotherapy and chemotherapy. Here, the authors suggest that hCINAP contributes to the resistance of acute myeloid leukemia cells by regulating SUMOylation of Nucleophosmin during the DNA-damage response.

## Introduction

Maintenance of genomic stability is critical for the proper functioning of organisms. The genome of a cell is continually under attack from various DNA-damaging agents, and double strand break (DSB) is the most deleterious of such events^1^. To maintain genomic stability following DSBs, cells have developed two major DSB-repair pathways, classical non-homologous end-joining (c-NHEJ), and homologous recombination (HR), both of which are indispensable to the DNA-damage response (DDR) system^1^. However, if damage to the DNA is too severe to repair, the cell will undergo apoptosis. To understand the mechanisms involved in the DDR, identification of regulatory proteins that participate in and control these repair pathways is of primary importance.

In response to DNA-damage, a series of repair proteins sequentially accumulate at the damaged site and function to induce signal transduction pathways that initiate the subsequent repair of DSB^2^. These proteins are finely modulated by dynamic and reversible posttranslational modifications. Protein modifications via phosphate and ubiquitin have already been recognized as important regulators of the DDR. Increasing evidence also indicates that SUMO is involved in the DNA-repair mechanisms of higher organisms^3^. During DNA-damage or replication stress, components of the SUMO pathway accumulate at DSB sites within the nucleus, including E1 SUMO-activating enzyme, E2-conjugating enzyme, and E3 SUMO ligases^4–6^. The reversibility of SUMOylation is conferred by the Sentrin/SUMO-specific proteases (SENPs)^7^. Previous studies have mainly focused on the role of the E2 Ubc9 and E3 PIAS family proteins^8^; however, the physiological role of SUMO proteases in the DSB pathway is not well understood. Numerous studies have attempted to explain how SUMOylation takes part in the DSB-induced DDR^5,9^. Moreover, deregulation of the SUMO pathway has been found to correlate with various cancers^10^. As such, direct targeting of SUMOylation could be helpful for the diagnosis, prognosis, and treatment of various cancers.

Acute myeloid leukemia (AML) is a serious hematological malignancy^11^. Nucleophosmin (NPM1) gene mutations represent the most-frequent genetic lesions in patients with AML^12^. The standard induction chemotherapy for AML relies on a combination of the nucleoside analog cytarabine (Ara-C) and an anthracycline, such as daunorubicin (DNR)^13^. Although most patients meet the remission expectations after initial chemotherapeutic treatment, relapse frequently occurs. As such, the global prognosis for these patients remains poor^6^. Generally, the mechanism of action of the chemotherapeutic drugs used for AML treatment relies on the inhibition of DNA synthesis and the induction of DNA DSB in cancer cells, eventually leading to cell apoptosis or cell death^14^. Very high rates of genomic instability and apoptosis have been associated with an improved prognosis in patients with AML. Importantly, induction of the DDR is one of the main consequences of taking these drugs, leading to the induction of DNA repair via the c-NHEJ and HR pathways. However, the cellular effectors influencing these two repair pathways in AML have not been clearly identified^15^.

Human coilin-interacting nuclear ATPase protein (hCINAP), also known as adenylate kinase 6, is highly conserved in eukaryotes^16,17^. In human cells, hCINAP participates in the formation of Cajal bodies^18^, affects p53 activity via the HDM2-p53 pathway^19^, regulates 18 S rRNA processing^20^, and determines self-renewal of colorectal cancer stem cells by modulating the Warburg effect^21^. However, the physiological function of hCINAP in the DDR and maintenance of genome stability has not been elucidated.

Here, we determine that hCINAP is involved in the relatively late stage of the DDR by inhibiting NPM1 SUMOylation in a SENP3-dependent manner. DNA-damage-induced BRCA1 recruitment and the choice of DNA repair pathways are fine-tuned by hCINAP. These findings suggest that hCINAP is a potential and promising target for overcoming resistance towards chemotherapy and radiotherapy in patients with AML.

## Results

### hCINAP is essential for genome stability and is associated with AML

**Fig. 1:**
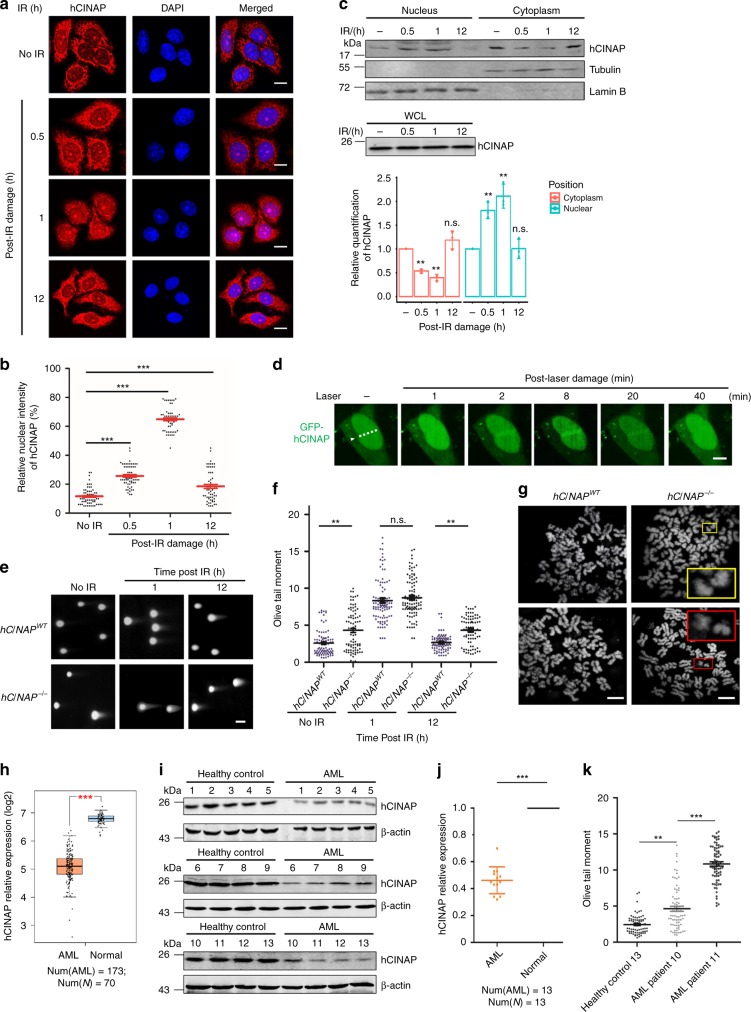
hCINAP is essential for genome stability and is associated with AML. **a** U2OS cells were treated with or without IR for the indicated times and immunostained with an anti-hCINAP antibody. Scale bar, 10 μm. **b** Quantification analysis of hCINAP nuclear intensity was normalized to that of DAPI using Volocity software. Statistical analysis is presented as the mean ± SEM, ***P < 0.001. More than 50 cells were counted per group. **c** U2OS cells were treated with 10 Gy IR and collected at the indicated time. The nucleus–cytoplasm fraction analysis was performed. The densitometry bar graph was generated by quantifying blots from three independent experiments using ImageJ. **d** A live cell imaging system was used to monitor the recruitment of GFP-hCINAP protein to DNA-damage sites. Scale bar, 10 μm. **e**, **f** *hCINAP*^*WT*^ and *hCINAP*^−/−^ HEK293T cells were treated with 10 Gy of irradiation and subjected to neutral comet assay. The olive tail moments were quantified using the Student’s *t* test; *P < 0.05, **P < 0.01. The knockout efficiency of hCINAP was confirmed in Supplementary Fig. 1b, c. **g** Examples of chromosome spreads are shown. Scale bar, 5 μm. Inset rectangles show *hCINAP*^−vbn/−^ chromosome fragments and chromatid breaks. A total of 2000 chromosomes for each cell line were karyotyped and chromosome abnormalities were detected. The statistical result is shown in Supplementary Fig. 1d. **h** Expression of hCINAP in AML patients relative to that of healthy controls was analyzed by using the TCGA database. **i**, **j** White blood cells of AML patients and healthy people were separated from the peripheral blood and hCINAP expressions were analyzed via immunoblotting. We compared the protein expression using a Wilcoxon matched pairs test. **k** White blood cells were subjected to the neutral comet assay to assess the effect of hCINAP on genomic stability. More than 100 cells were counted in each group. The quantified data are shown (the representative images are shown in Supplementary Fig. 1e). Statistical analysis was performed using Student’s *t* test (**P < 0.01; ***P < 0.001). The source data can be found in Supplementary Data 1. Unprocessed scans of blots are provided in Supplementary Fig. 13

To explore whether hCINAP is involved in the DDR, U2OS cells were treated with ionizing radiation (IR) to induce DSBs. In response to damage stimuli, hCINAP translocated from the cytoplasm to the nucleus in immunofluorescence assays (Fig. 1a, b). Consistent with these observations, nuclear hCINAP increased, whereas cytoplasmic hCINAP decreased following IR treatment (Fig. 1c). These results indicated that hCINAP functions in a spatiotemporal manner in the process of DNA repair. Next, a laser micro-irradiation system was utilized to generate localized DNA-damage in GFP-hCINAP expressing U2OS cells. GFP-hCINAP was recruited to the laser-induced DNA-damage tracks after micro-irradiation. The fluorescent signals began to intensify ~ 2 min after micro-irradiation, reaching a maximum after ~ 8 min, and decreased thereafter, receding to pre-damage levels by ~ 40 min after irradiation (Fig. 1d), suggesting that the recruitment of hCINAP to DNA-damage sites was a chronological process. The recruitment of endogenous hCINAP to the damaged sites was also detected (Supplementary Fig. 1a). We deduced that hCINAP does not function during the relatively early damage response, as it is recruited to DNA-damage sites after a subsequent lag period following irradiation.

In addition, we constructed an *hCINAP*^−/−^ cell line using the CRISPR/Cas9 system (Supplementary Fig. 1b, c) and evaluated the global effects of hCINAP on DNA-damage via neutral comet assays. Knockout of hCINAP caused the appearance of tails, photocopying DNA damage caused by genome instability (Fig. 1e, f). The *hCINAP*^−/−^ cells showed higher basal DNA-damage levels than wild-type cells. Similar comet tail length was observed 1 h after irradiation between the two groups. However, in the relatively later stage, after a 12-hour recovery following irradiation, *hCINAP*^−/−^ cells still had longer tails than wild-type cells. Furthermore, hCINAP-depleted cells accumulated chromosome breaks and showed chromosome instability phenotypes (Fig. 1g). Loss of *hCINAP* in cells induced a higher frequency (5.65%) of chromosome rearrangements compared with the 2.87% total breaks per chromosome in hCINAP wild-type cells (Supplementary Fig. 1d), which is similar to that of p53 reported previously^22^. Collectively, these results indicate that hCINAP functions at a relatively late stage in the DDR pathway and is essential for maintaining genome stability.

AML is a serious hematological malignancy with well-known radiotherapy and chemotherapy resistance, and high rates of genomic instability in AML cells have been associated with improved prognosis in patients with AML^11^. Considering the indispensable role of hCINAP in maintaining genomic stability, we wanted to investigate whether hCINAP expression affects AML diagnosis and therapy. Using the TCGA and GTEx databases, we observed that hCINAP expression levels were frequently downregulated in AML compared with healthy controls (Fig. 1h). We collected the peripheral blood (PB) of patients with AML and healthy controls without any sign of hematological malignancies and detected low expression levels of hCINAP in AML patients (Fig. 1i, j). To verify the role of hCINAP in maintaining genomic stability, we performed neutral comet assays on three samples: healthy control 13 with the highest hCINAP expression level, AML 10 with moderate hCINAP expression, and AML 11 with the lowest level of hCINAP expression. As expected, healthy control 13 had the lowest rate of genomic instability, whereas the highest genomic instability frequency was observed in AML sample 11 (Fig. 1k, Supplementary Fig. 1e). These results support the observation that hCINAP is essential for genomic stability. Furthermore, we detected chromosome morphology abnormalities, using a metaphase spread assay, in PB cells from healthy control 13, AML 10, and AML 11 (Supplementary Fig. 1f). Low hCINAP expression in PB cells from AML patients induced a higher frequency of chromosome rearrangements. The AML PB cells and KG-1α cells with lower abundance of hCINAP accumulated more chromosome breaks and showed more chromosome instability phenotypes (Supplementary Fig. 1e–g). The total RNA from *hCINAP*^*WT*^ and *hCINAP*^−/−^ U2OS cells was extracted for RNA-seq analysis. KEGG pathway enrichment analysis showed that *hCINAP* is truly related to hematological diseases (Supplementary Fig. 1h). Collectively, these results demonstrate that the necrotic white cells from AML samples had lower levels of hCINAP and lower genomic stability and were, thus, highly sensitive to DNA-damage stimuli.

### NPM1 is a partner protein of hCINAP

**Fig. 2:**
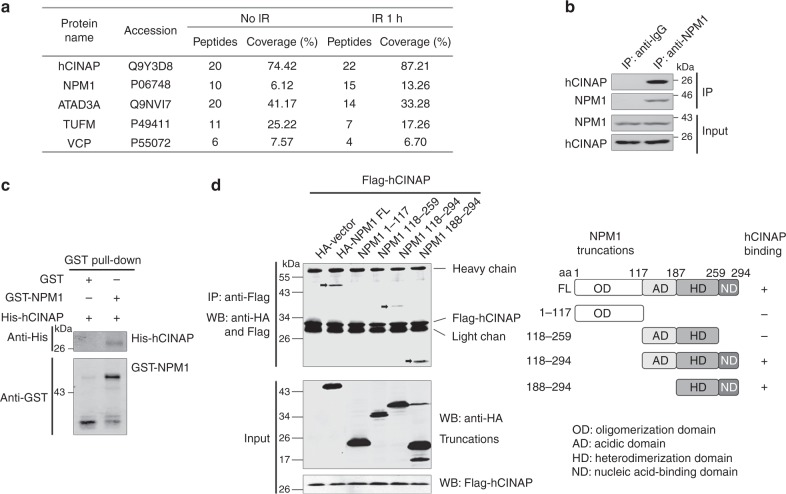
NPM1 is a new partner protein of hCINAP. **a** HEK293T cells harboring the Flag-empty vector or Flag-hCINAP were treated with or without IR (6 Gy) and then lysed and subjected to affinity purification using anti-Flag M2 affinity beads. The purified protein complex was analyzed by mass spectrometry. The major hits from mass spectrometry are shown in the table. **b** The interaction between endogenous hCINAP and NPM1 in HEK293T cells was confirmed by co-IP assays. Whole-cell lysates were immunoprecipitated with an antibody against NPM1 followed by immunoblotting using the indicated antibodies. **c** His-hCINAP and GST-NPM1 proteins were purified from *E. coli*, and in vitro pull-down analysis was performed using the GST protein as a negative control. **d** Mapping of the interaction domain of NPM1 with hCINAP. HEK293T cells expressing the Flag-hCINAP, HA-NPM1 WT, or NPM1 truncated mutants were subjected to the co-IP assays. A schematic of the different truncated mutants of NPM1 is shown. The source data can be found in Supplementary Data 1. Unprocessed scans of blots are provided in Supplementary Fig. 13

To elucidate the underlying mechanism of hCINAP in the regulation of the DDR, we attempted to identify proteins that were associated with hCINAP in vivo via immunoprecipitation (IP) followed by mass spectrometry analysis. The major hits from the mass spectrometry analyses are shown in Fig. 2a. Among these proteins, NPM1 had a strong interaction with hCINAP. NPM1 has a crucial role in the regulation of cell growth, proliferation, and transformation^23^ and is one of the most frequent targets of genetic alterations in hematopoietic tumors^24^. Subsequently, we confirmed the interaction between hCINAP and NPM1 by both co-immunoprecipitation (co-IP) and in vitro GST pull-down experiments (Fig. 2b, c). The interaction between endogenous hCINAP and NPM1 was confirmed in the NPM1 WT OCI-AML2 cell line (Supplementary Fig. 2a) and NPM1 mutant OCI-AML3 cell line (Supplementary Fig. 2b). We also determined that the C-terminal nucleic-acid-binding domain was critical for its binding to hCINAP (Fig. 2d). Collectively, these data demonstrated that hCINAP direct interacts with NPM1.

### hCINAP regulates NPM1 deSUMOylation during DSB repair

**Fig. 3:**
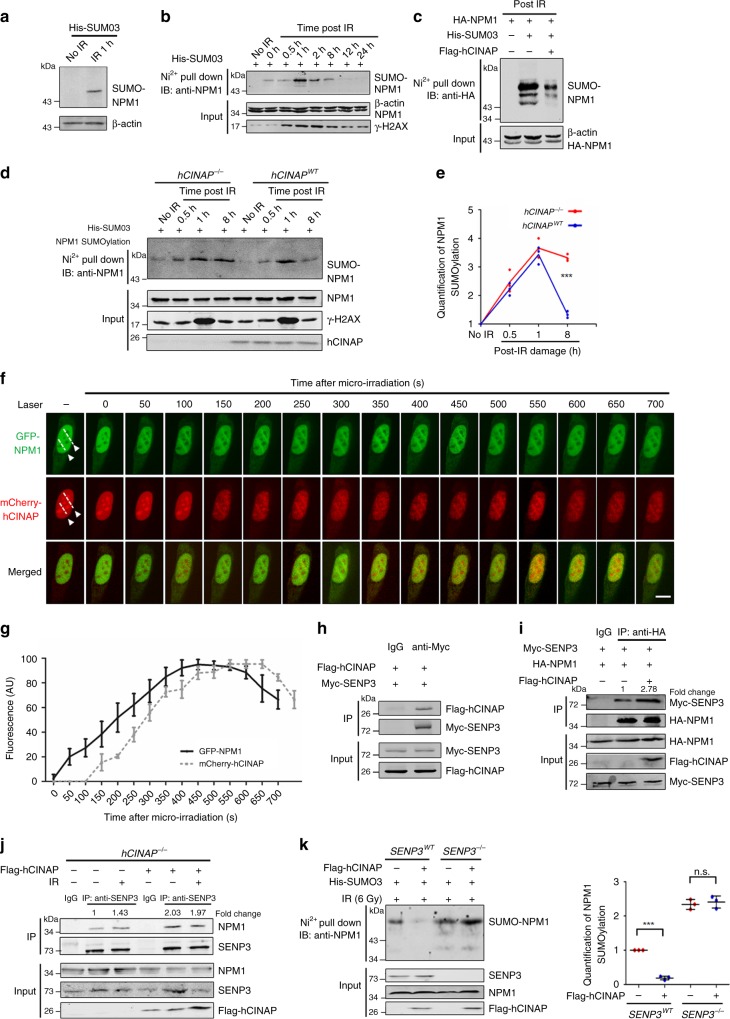
hCINAP regulates NPM1 deSUMOylation in a SENP3-dependent manner during DSB repair. **a**, **b** The effect of damage on NPM1 deSUMOylation was investigated using a His-SUMO pull-down assay. Nickel precipitation (Ni^2+^) from His-SUMO3-expressing U2OS cells treated with IR at different time points is shown. The level of NPM1 SUMOylation was assessed using an anti-NPM1 antibody. **c**, **d** *hCINAP*^*WT*^ and *hCINAP*^−/−^ HEK293T cells were transfected with His-SUMO3 for 48 h and then treated without or with IR (10 Gy). Cells were collected at different time points after IR treatment (**c**, 8 h; **d**, a time course is shown in the Fig.) and subjected to His-SUMO pull-down analysis to access the level of NPM1 SUMOylation. **e** The densitometry analysis was done on three independent His pull-down experiments of Fig. 3d. **f**, **g** Dynamic accumulation of NPM1 and hCINAP at DNA-damage sites in living cells. U2OS cells expressing GFP-hCINAP and mCherry-hCINAP were monitored after micro-irradiation via time-lapse fluorescence microscopy **f**. Quantifications of GFP-hCINAP and mCherry-hCINAP accumulation at laser track sites were performed using ImageJ software **g**. The fluorescence intensity values in the micro-irradiated areas were pooled from 10 independent cells and plotted vs. time. **h** HEK293T cells were transfected with Myc-SENP3 and Flag-hCINAP for 48 h. Co-IP assays were then performed using an anti-Myc antibody. The bound complexes were analyzed via immunoblotting. **i** HEK293T cells were transfected with the indicated plasmids. Cell lysates were subjected to co-IP analyses to examine the effects of hCINAP on the interaction between SENP3 and NPM1 using the indicated antibodies. **j** *hCINAP*^−/−^ HEK293T cells were transfected with the indicated plasmids. Cell lysates were subjected to co-IP analyses to examine the effects of hCINAP on the interaction between SENP3 and NPM1 using the indicated antibodies. **k** The *SENP3*^−/−^ cell line was constructed using the CRISPR/Cas9 system as shown in Supplementary Fig. 2c. Knockout efficiency was determined by immunoblotting using an anti-SENP3 antibody (Supplementary Fig. 2d). The level of endogenous NPM1 SUMOylation was monitored via His-SUMO pull-down analysis in *SENP3*^*WT*^ and *SENP3*^−/−^ cells. The densitometry analysis was carried out on three independent His pull-down experiments. Unprocessed scans of blots are provided in Supplementary Fig. 13

As hCINAP interacts with NPM1, we examined how hCINAP regulates NPM1 in DSB. First, we found that overexpression of hCINAP did not significantly affect NPM1 expression (Supplementary Fig. 3a). A previous study showed that NPM1 is modified by phosphoric acid, ubiquitin, and SUMO chains^25^. Therefore, we tested whether hCINAP could affect such modifications in relation to NPM1. Overexpression of hCINAP did not affect NPM1 phosphorylation levels (Supplementary Fig. 3a). His-ubiquitin pull-down analysis also showed that hCINAP was dispensable for NPM1 ubiquitination (Supplementary Fig. 3b). His-SUMO pull-down assays, however, indicated that endogenous NPM SUMOylation could be detected only when the damage occurred, and this changed dynamically during the DDR (Fig. 3a, b). The SUMOylation of endogenous NPM1 increased in response to damage and reached its peak at 1 h after irradiation. After 1 h, the level of NPM1 SUMOylation decreased and was barely detectable 12 h after irradiation (Fig. 3b). In the post IR period, such as 8 h after IR treatment, hCINAP obviously inhibited IR-induced NPM1 SUMOylation (Fig. 3c), wheresas in *hCINAP*^−/−^ cells, the level of NPM1 SUMOylation was maintained at a high level, especially in the late DDR stage (8 h after IR) (Fig. 3d, e). These results indicate that the increased IR-induced NPM1 SUMOylation cannot return to its basal level in the absence of *hCINAP*.

UV treatment has been shown to promote NPM1 phosphorylation^26^. Thus, we investigated the cross-talk between different NPM1 posttranslational modifications. We overexpressed hCINAP with either a SUMOylation deficient mutation (K263R) or a phosphorylation deficient mutation (T199A) and detected phosphorylation or SUMOylation changes, respectively (Supplementary Fig. 3c–e). We observed no cross-talk between NPM1 SUMOylation and phosphorylation. Therefore, in the following studies, we mainly focused on the function of hCINAP with regard to the regulation of NPM1 SUMOylation and its association within the DDR. We assessed the timing of recruitment of these two proteins to damage sites following micro-irradiation. NPM1 accumulated at irradiated sites within 10 s after micro-irradiation, whereas hCINAP accumulated at irradiated sites within 2 minutes after micro-irradiation (Fig. 3f, g), suggesting that NPM1 and hCINAP may function at different time points during the DDR process.

### hCINAP-regulated NPM1 deSUMOylation depends on SENP3

As hCINAP is an adenylate kinase but not a SUMO protease^16–18^, we questioned how hCINAP could inhibit NPM1 SUMOylation. To assess the involvement of the enzymatic activity of hCINAP in NPM1 deSUMOylation, his pull-down assays were performed with the enzymatic mutants, hCINAP-D77G and hCINAP-H79G (Supplementary Fig. 3f). Both wild-type hCINAP and hCINAP-H79G and D77G mutants reduced IR-induced NPM1 SUMOylation, suggesting that hCINAP-mediated deSUMOylation of NPM1 does not depend on hCINAP enzymatic activity. SENP3 is reported to be a specific protease inhibiting NPM1 SUMOylation^10^. First, by co-IP analysis, we showed that hCINAP clearly binds to SENP3 (Fig. 3h, Supplementary Fig. 2a, b). Moreover, hCINAP enhanced the interaction between NPM1 and SENP3 (Fig. 3i, j). These observations indicate that hCINAP promotes desumoylation of NPM1 by the protease SENP3.

Next, we explored whether the inhibition of NPM1 SUMOylation by hCINAP was SENP3 dependent. We used the CRISPR/Cas9 system to construct *SENP3*^−/−^ cell lines (Supplementary Fig. 2c, d) and compared the inhibitory effect of hCINAP on NPM1 SUMOylation in *SENP3*^−/−^ cells and *SENP3*^*WT*^ cells. In the DNA-damaged state, when SENP3 was knocked out, hCINAP did not inhibit NPM1 SUMOylation (Fig. 3k). Moreover, neutral comet assays showed that *SENP3* was also essential for genomic stability. When *SENP3* was knocked out, the tail of the comet was much longer than that of wild type (Supplementary Fig. 2e). Taken together, these results suggest that hCINAP inhibits NPM1 SUMOylation in a SENP3-dependent manner.

### NPM1 SUMOylation promotes DSB-induced BRCA1 accumulation

Based on the above discoveries that NPM1 SUMOylation is correlated with the occurrence of DSBs and rapidly increased following DNA damaging stimuli, we questioned whether NPM1 SUMOylation was necessary for the dynamic regulation of the recruitment of DNA-damage proteins. NPM1 phosphorylation has been shown to influence H2A ubiquitination and RNF8, RNF168 recruitment^26^. We found that NPM1 and its SUMOylation showed no effect on H2A ubiquitination (Supplementary Fig. 4a). Moreover, no significant changes in the foci formation of MDC1, RNF8, RNF168, and FK2 were observed in the NPM K263R mutant or hCINAP-deficient cell lines (Supplementary Fig. 4b–e and Fig. 5a-d). These data indicate that NPM1 SUMOylation does not influence H2A ubiquitination and subsequent recruitment of the DNA-damage repair proteins.

**Fig. 4:**
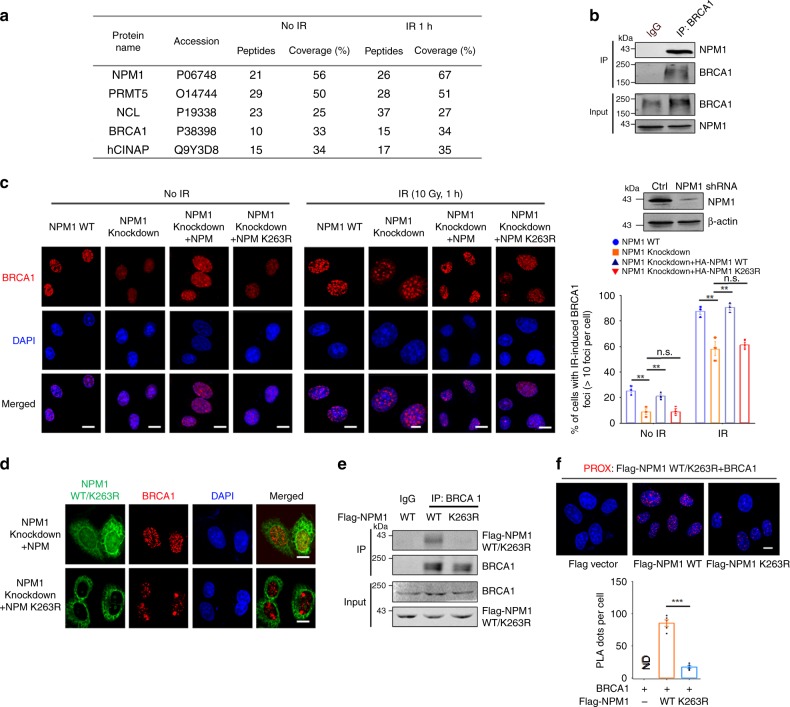
NPM1 SUMOylation promotes DSB-induced BRCA1 accumulation. **a** HEK293T cells harboring the Flag-empty vector or Flag-NPM1 were treated with or without IR (6 Gy) and then lysed and subjected to affinity purification using anti-Flag M2 affinity beads. The purified protein complex was analyzed via mass spectrometry. The major hits from mass spectrometry are shown in the table. **b** The interaction between NPM1 and BRCA1 was confirmed using a Co-IP assay with the indicated antibodies. **c** Immunofluorescence assays to analyze the effect of NPM1 SUMOylation on IR-induced BRCA1 foci. U2OS cells with depleted NPM1 were rescued with or without wild-type NPM1 or the NPM1 K263R mutant and treated with or without IR (10 Gy). Representative images are shown and the percentage of cells with more than 10 BRCA1 foci were counted. Scale bar, 10 μm. The results are presented as the mean ± SEM of three biological replicates. Statistical analysis was performed using the Student’s *t* test; **P < 0.01, ***P < 0.001. Approximately 100 cells in each group were counted. **d** HeLa cells infected with lentivirus expressing NPM1 shRNA were transfected with wild-type NPM1 or K263R mutant. After transfection, cells were exposed to 6 Gy IR and then recovered for 1 h before being subjected to immunofluorescence using antibodies against NPM1 and BRCA1. Representative NPM1 WT/K263R, BRCA1 foci, and DAPI-stained nuclei are shown. Scale bar, 10 μm. **e** HEK293T cells transfected with Flag-NPM1 WT or K263R mutant vectors were subjected to co-IP analysis using the indicated antibodies. **f** U2OS cells transfected with Flag-NPM1 WT or K263R were grown on collagen-coated microchamber slides. After fixation, the interaction between BRCA1 and NPM1 WT or K263R was detected by in situ PLA using anti-Flag and anti-BRCA1 antibodies. The PLA-detected proximity (PROX) complexes are represented by the fluorescent rolling circle products (red dots). Scale bar, 10 μm. Quantification of the PROX dots per cell is shown as mean ± SEM with the *P* value indicated. ***P < 0.001. The source data can be found in Supplementary Data 1. Unprocessed scans of blots are provided in Supplementary Fig. 13

We then identified repair proteins including BRCA1 that were potentially associated with NPM1 (Fig. 4a). Co-IP assays demonstrated that endogenous NPM1 indeed interacted with BRCA1 in vivo (Fig. 4b). To determine whether NPM1 and its SUMOylation were important for the recruitment of repair proteins, we constructed an NPM1 knockdown U2OS cell line and examined the formation of BRCA1 foci at different time lags following IR treatment. NPM1 depletion reduced the recruitment of BRCA1 to the damage sites and could be rescued by introduction of wild-type NPM1, but not the NPM1 K263R mutant, a mutant dysfunctional at the major SUMOylation catalytic site (Fig. 4c). Similar changes in BRCA1 foci were observed at 1 h after IR treatment. We further confirmed the role of NPM1 SUMOylation in the formation of BRCA1 foci and the interaction between NPM1 and BRCA1 by immunofluorescence and co-IP experiments. The NPM1 K263R mutant no longer localized to the nucleus and, consequently, the number of IR-induced BRCA1 foci decreased significantly in comparison to NPM1 WT cells (Fig. 4d). Meanwhile, BRCA1 only interacted with NPM1 WT but not NPM1 K263R (Fig. 4e). Moreover, a proximity ligation assay (PLA), which enables detection of protein interaction in situ with high specificity and sensitivity^27,28^, was performed showing that NPM1 WT and BRCA1 interacted mainly in the nucleus (Fig. 4f). These results indicate that NPM1 SUMOylation is necessary for the interaction between NPM1 and BRCA1 and promotes the accumulation of BRCA1 foci induced by DSB.

### Loss of hCINAP leads to defects in error-free DSB repair HR

**Fig. 5:**
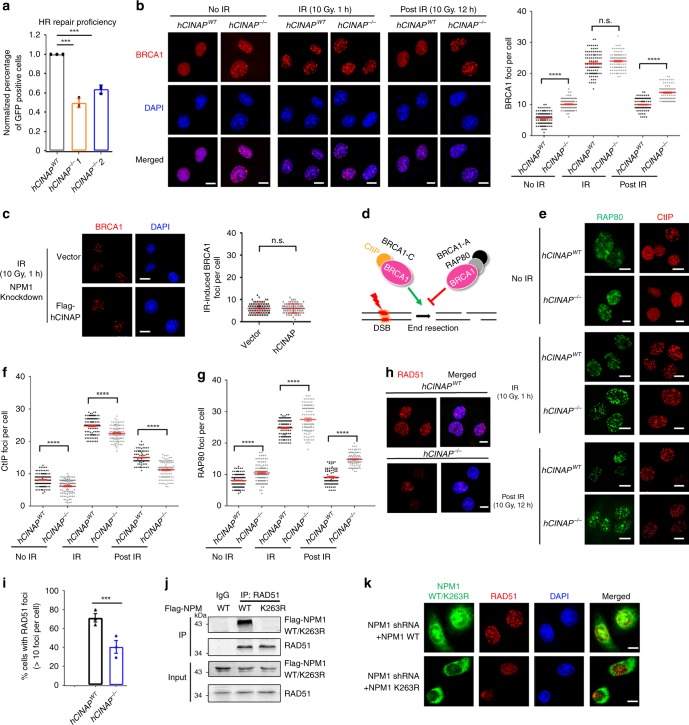
Loss of hCINAP leads to a defect in error-free HR pathway. **a** *hCINAP*^*WT*^ and *hCINAP*^−/−^ HEK293T cells were subjected to HR assays. The data are presented as the mean ± SEM of three replicates. More than 1000 cells were counted in each group, two-tailed students’ *t* test, ***P < 0.001. **b** *hCINAP*^*WT*^ and *hCINAP*^−/−^ U2OS cells treated with or without IR (10 Gy) were subjected to immunofluorescence assays. Representative immunofluorescence images are shown (left), and statistics of BRCA1 foci numbers in each group are shown (right). ****P < 0.0001. **c** NPM1 knockdown U2OS cells transfected with the indicated plasmids were treated with IR (10 Gy) and released for 1 h. The accumulation of BRCA1 was then assessed. Scale bar, 10 μm. **d** Schematic of the role of different BRCA1 complex in end resection. **e**–**g** *hCINAP*^*WT*^ and *hCINAP*^−/−^ U2OS cells were immunostained with anti-RAP80 or anti-CtIP antibodies. Representative immunofluorescence images are shown in **e**, and BRCA1 foci numbers in cells were counted in **f** and **g**. Scale bar, 10 μm. ****P < 0.0001. **h**, **i** Immunofluorescence **h** and its quantification **i**showing that *hCINAP* deficiency blocked the RAD51 foci recruitment. Representative images and percentages of cells with > 10 RAD51 foci were counted. The U2OS cells were treated with 4 Gy IR and released 4 h before fixing. Scale bar, 10 μm. ***P < 0.001. **j** HEK293T cells transfected with the indicated vectors were subjected to co-IP analysis using the indicated antibodies. **k** Cells were exposed to 10 Gy IR and then recovered for 1 h before being subjected to immunofluorescence. Scale bar, 10 μm. Additional cell images and quantification of RAD51 foci are shown in Supplementary Fig. 12. For **b**, **c**, **f**, **g**, the results are shown as the mean ± SEM from three independent experiments. Data were analyzed using Student’s *t* test. More than 100 cells were counted per group. Unprocessed scans of blots are provided in Supplementary Fig. 13

As HR and NHEJ are the two main DSB repair pathways^1^, we next evaluated the effects of hCINAP on these pathways. Depletion of hCINAP resulted in a significant decrease in the percentage of GFP-positive cells (Fig. 5a), demonstrating that loss of hCINAP function impairs the proficiency of error-free HR repair. We assessed the abundance of BRCA1 foci in *hCINAP*^*WT*^ and *hCINAP*^−/−^ U2OS cells at different time points following IR treatment. The *hCINAP*^−/−^ U2OS cells had much more BRCA1 foci than *hCINAP*^*WT*^ cells in the resting state. After exposure to irradiation, BRCA1 foci in *hCINAP*^−/−^ cells were more abundant than in *hCINAP*^*WT*^ cells. At 1 h after irradiation, *hCINAP*^−/−^ cells contained slightly more BRCA1 foci at the lesion points than *hCINAP*^*WT*^ cells, suggesting that hCINAP moderately regulates IR-induced DNA-damage foci during the early DNA repair period. Strikingly, after 12 h of recovery, *hCINAP*^−/−^ cells still had much more BRCA1 foci than *hCINAP*^*WT*^ cells (Fig. 5b), suggesting that hCINAP affects the dissociation of BRCA1 from the lesion sites at a relatively late stage after damage repair.

To verify the regulation of BRCA1 recruitment in AML, we isolated white cells from the PB of AML patients and knocked down hCINAP expression. We then examined the effect of hCINAP depletion on BRCA1 foci formation. Knockdown of hCINAP increased the number of BRCA1 foci in leukocytes isolated from AML patients (Supplementary Fig. 6a, b). Collectively, these results indicate that hCINAP affects BRCA1 foci dissociation from lesion points in the DSB repair process, especially in the relatively late stage.

We next investigated whether hCINAP-regulated BRCA1 foci recruitment via its regulation of NPM SUMOylation. Indeed, when NPM1 was knocked down, BRCA1 foci remained at very low levels even in the damaged state. In addition, hCINAP no longer influenced BRCA1 foci recruitment (Fig. 5c). These results indicate that hCINAP inhibits BRCA1 recruitment in the damaged state in an NPM1-dependent manner.

Normally, the recruitment of BRCA1 is considered a landmark event in the HR pathway^5^. However, the data above showed that depletion of hCINAP reduced HR efficiency but increased the abundance of BRCA1 foci. To explain this seemingly controversial observation, we further examined the effects of hCINAP on foci formation of RAP80 and CtIP. RAP80 and CtIP are the key factors in the BRCA1-A and BRCA1-C complexes, respectively^29^. As indicated in Fig. 5d, the formation of the BRCA1-C complex containing CtIP and BRCA1 promotes end resection and the subsequent process of HR, whereas the Abraxas/RAP80/BRCA1-containing BRCA1-A complex has the opposite effect on HR^29^. Consistent with the pattern in Fig. 5d, knockdown of hCINAP increased RAP80 foci recruitment but reduced CtIP foci (Fig. 5e–g). These observations indicate that hCINAP regulates the end resection process by selectively affecting the repair proteins RAP80 and CtIP of the BRCA1 complexes, thus affecting the efficiency of HR.

RAD51 has a central role in the HR pathway. After a DSB occurs, RAD51 promotes invasion of ssDNA into undamaged homologous dsDNA^30^. We further assessed the role of hCINAP in the end resection process by examining its influence on IR-induced RAD51 protein recruitment. Deficiency of hCINAP blocked RAD51 foci formation (Fig. 5h, i), which is consistent with its effect on HR repair efficiency (Fig. 5a). Furthermore, we found that RAD51 only bound to NPM1 WT but not NPM1 K263R (Fig. 5j). Thus, in NPM1 K263R cells, the number of IR-induced RAD51 foci decreased significantly (Fig. 5k, Supplementary Fig. 12). These results indicate that NPM1 SUMOylation promotes RAD51 recruitment.

CHK1 is one of the ATR substrates, and CHK1 phosphorylation is critical for end resection. We next examined the effect of NPM1 SUMOylation on CHK1 phosphorylation. Comparison of the abundance of p-CHK1 in K263R mutant cells with that of NPM1 WT cells revealed that IR treatment promoted CHK1 phosphorylation, which was inhibited by NPM1 knockdown. Furthermore, re-expression of NPM1 WT, but not the K263R mutant, significantly attenuated the abundance of p-CHK1 (Supplementary Fig. 7a), suggesting that only NPM1 WT could rescue the reduced IR-induced CHK1 phosphorylation. Consistent with this data, measurement of HR efficiency showed that endogenous NPM1 expression and its SUMOylation are necessary for efficient HR repair (Supplementary Fig. 7b). Taken together, hCINAP functions in the HR pathway through regulation of NPM1 SUMOylation.

### SUMOylated NPM1 directly binds to RAP80

**Fig. 6:**
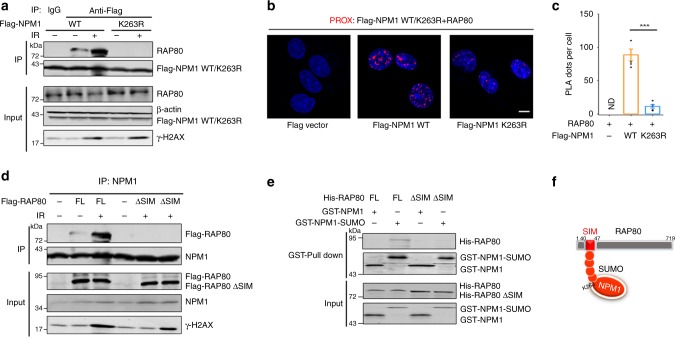
SUMOylated NPM1 directly binds to the RAP80 SUMO-interacting motif (SIM) domain. **a** HEK293T cells transfected with Flag-NPM1 WT or K263R mutant vectors were treated with or without IR, and then subjected to co-IP analysis using anti-RAP80 and anti-Flag antibodies. **b** U2OS cells transfected with Flag-NPM1 WT or K263R were grown on collagen-coated microchamber slides. After fixation, in situ PLA for NPM1 WT or K263R and RAP80 was performed with anti-Flag and anti-RAP80 antibodies. The PLA-detected proximity (PROX) complexes are represented by the fluorescent rolling circle products (red dots). Scale bar, 10 μm. **c** Quantification of the PROX dots per cell is shown as mean ± SEM with the *P* value indicated. ***P < 0.001. **d** HEK293T cells transfected with Flag-RAP80 FL or ΔSIM mutant vectors were treated with or without IR, and then subjected to co-IP analysis using anti-NPM1 and anti-Flag antibodies. **e** In vitro interaction of purified RAP80 with NPM1-SUMO2/3. The full-length and SIM domain (40–47 aa) deleted truncation of His-RAP80 and GST-NPM1 were purified from *E. coli*. GST-NPM1 protein was SUMOylated in vitro, and the pull-down analysis was performed. **f** Schematic of the direct interaction between SUMOylated NPM1 and RAP80 SUMO-interacting motif (SIM). Unprocessed scans of blots are provided in Supplementary Fig. 13

Considering NPM1 SUMOylation is beneficial for the recruitment of repair proteins, such as RAP80 (Fig. 5d), we next investigated if NPM1 SUMOylation is required for recruitment of RAP80. In NPM1-depleted cells, we reintroduced NPM1 WT or the NPM1 K263R mutant, and found that IR treatment enhanced the interaction between wild-type NPM1 and RAP80, whereas NPM1 K263R mutant showed no interaction with RAP80 (Fig. 6a). PLA analysis also showed increased interacting PLA signals in cells with NPM1 WT and RAP80 compared with cells with NPM1 K263R-RAP80 (Fig. 6b, c). As SUMO and UB modifications are coordinated to recruit RAP80 and BRCA1 to DNA-damage sites^31,32^, we constructed two expression vectors, RAP80 full-length (FL) and SIM domain-deleted RAP80 (ΔSIM), and examined whether SUMOylated NPM1 directly interacted with the SIM domain of RAP80 in vivo and in vitro. Deletion of the SIM domain abrogated the interaction between NPM1 and RAP80 with/without IR (Fig. 6d). Consistently, GST-NPM1-SUMO2 could directly bind to His-RAP80 FL but not His-RAP80 ΔSIM, and no interaction was observed between unSUMOylated GST-NPM1 and His-RAP80 FL or His-RAP80 ΔSIM (Fig. 6e). These data suggest that SUMOylated NPM1 directly interacts with the SIM domain of RAP80 (Fig. 6f). These results indicate that the SIM domain of RAP80 is required for its interaction with NPM1 and NPM1 SUMOylation is essential for recruitment of RAP80.

### Deletion of hCINAP increases NHEJ efficiency

**Fig. 7:**
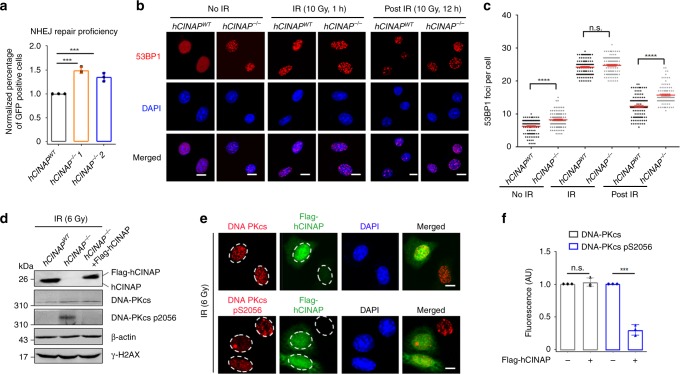
Deletion of hCINAP increases the efficiency of error-prone NHEJ. **a** *hCINAP*^*WT*^ and *hCINAP*^−/−^ HEK293T cells were subjected to NHEJ assays. Data are presented as the mean ± SEM of three biological replicates. More than 1000 cells were counted in each experimental group. The positive cell percentage was compared with the control group, two-tailed students’ *t* test, **P < 0.01, ***P < 0.001. **b**, **c** *hCINAP*^*WT*^ and *hCINAP*^−/−^ U2OS cells were subjected to 10 Gy IR and immunostained with anti-53BP1 antibodies. Representative immunofluorescence images are shown in **b** and the statistical analysis of 53BP1 foci in each group were counted **c**. The results are presented as the mean ± SEM of three biological replicates. Statistical analysis was performed using the Student’s *t* test; ****P < 0.0001. Approximately, 100 cells in each group were counted. **d** Different HEK293T cells were exposed to 6 Gy IR and recovered for 1 h, and then the levels of DNA-PKcs and phosphorylated DNA-PKcs Ser2056 were assessed by immunoblotting using the indicated antibodies. **e**, **f** HeLa cells transfected with Flag-empty vector or Flag-hCINAP were treated with IR (6 Gy) and recovered for 1 h, and then immunofluorescence assays were performed using anti-DNA-PKcs, anti-phosphor-DNA-PKcs Ser2056, and anti-Flag antibodies. Scale bar, 10 μm. Quantification analysis was performed using ImageJ software. Statistical analysis was performed using Student’s *t* test (***P < 0.001). About 50 cells were counted in each group. Unprocessed scans of blots are provided in Supplementary Fig. 13

We next assessed the effect of hCINAP on the NHEJ repair pathway. Knockout of hCINAP was associated with a dramatic upregulation in the relative percentage of GFP-positive cells by about 45% (Fig. 7a). Furthermore, we elucidated the effect of hCINAP on the dynamic foci formation of 53BP1 and DNA-PKcs, two specific markers for NHEJ repair. The *hCINAP*^−/−^ cells had more 53BP1 foci than wild-type cells in the resting state. At 1 h after IR treatment, the number of 53BP1 foci increased similarly in both wild-type and *hCINAP*^−/−^ cells. When the cells were treated with IR followed by a 12-hour recovery, *hCINAP*^−/−^ cells presented with more 53BP1 foci (Fig. 7b, c). These results indicate that hCINAP inhibits 53BP1 foci dissociation in the post IR stage.

Consistent with this finding, we also found that knockout of hCINAP had no influence on the total protein level of DNA-PKcs, but promoted its Ser2056 phosphorylation. Re-expression of hCINAP rescued the inhibitory effect on DNA-PKcs phosphorylation (Fig. 7d). This result was confirmed by IR-induced immunofluorescence foci quantification (Fig. 7e, f). These results demonstrate that loss of hCINAP impaired HR proficiency, which was compensated by an increase in NHEJ repair. Together these results suggest that hCINAP functions in the efficient repair of DSBs.

### AML PDX mice with depleted hCINAP show higher drug sensitivity

**Fig. 8:**
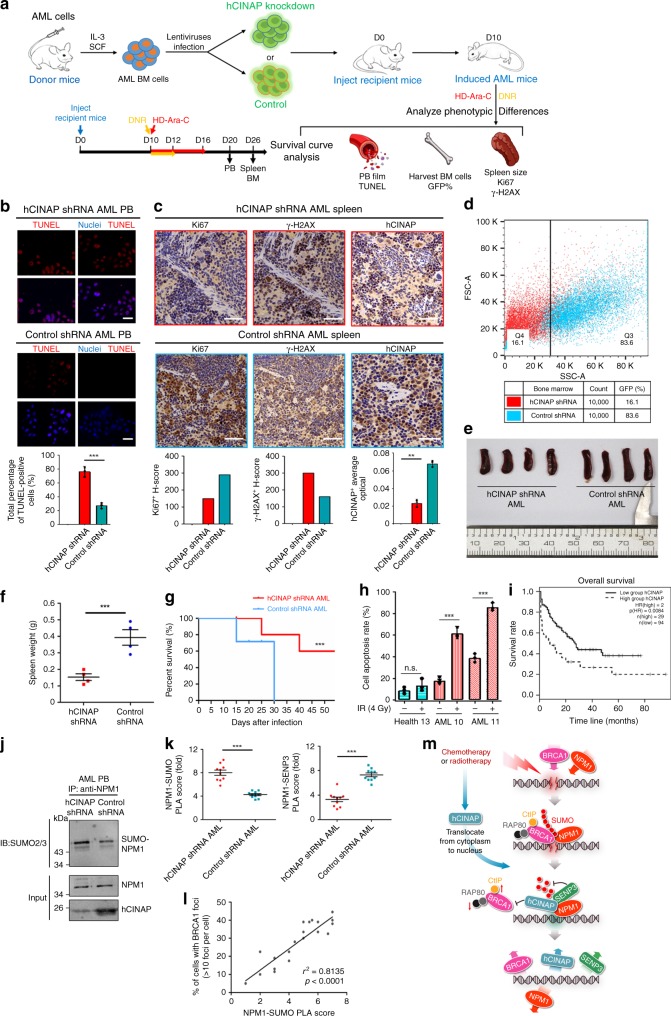
AML PDX mice with depleted hCINAP exhibit higher drug sensitivity. **a** Experimental design for generation of the hCINAP-depleted PDX AML mice models. **b**, **c** Representative staining of PB **b** at day 20 and spleen **c** at day 26 from AML mice. Scale bars, 50 μm (spleen Ki67, γ-H2AX, and PB TUNEL), 80 μm (splenic white pulp hCINAP). Histograms show the IHC staining quantification of Ki67 + , γ-H2AX^+^, and hCINAP expression (*n* = 2 for each group). Expression levels were quantified by the average optical density (AOD) of the positively stained cells with Image-Pro Plus 6.0 in spleens, which were measured by ImageJ. **d** Representative FACS plots show GFP-positive cells in the bone marrow of mice. *n* = 2 for each group. **e**, **f** Spleens of mice were weighed and photographed at the end of the study. Data were analyzed using Mann–Whitney test. **g** Kaplan–Meier survival curves of AML mice are shown. hCINAP shRNA, *n* = 12; Control shRNA, *n* = 12, ***P < 0.001. **h** Comparison of the apoptosis rates among white blood cells from AML patients 10 and 11 and control 13 shown in Fig. 1i. Apoptosis analyses were performed and > 10^5^ cells were counted in each group. **i** Kaplan–Meier analysis of overall survival of patients in the TCGA AML database. The solid and dashed lines represent the low and high expression group, respectively. The difference in the overall survival between these two groups was determined using a log-rank test. **j** Mice PB cells were collected from the orbit, and the leukocyte were harvested and dissociated. IP assay was done using anti-NPM1 antibody and the NPM1 SUMOylation level was determined by immunoblot using anti-SUMO2/3 antibody. **k** Quantified NPM1-SUMO PLA score (Left panel) and NPM1-SENP3 PLA score (Right panel) of spleen specimens from the two group of AML mice (*n* = 2). The representative images are shown in Supplementary Fig. 9a, b. **l** Scatterplot showing the positive correlation between PLA scores and BRCA1 foci in both AML mice groups. Pearson’s coefficient tests were performed to assess statistical significance. **m** Working model of hCINAP-mediated chemotherapy and radiotherapy resistance in the DDR. Unprocessed scans of blots are provided in Supplementary Fig. 13

The above data indicate that hCINAP is essential for maintaining genomic stability, and its expression level is associated with the prognosis of patients with AML (Fig. 1h–k). Thus, we explored its biological significance by constructing patient-derived xenograft (PDX) AML mice models with depleted hCINAP or wild-type hCINAP (Fig. 8a). PB leukocyte cells from AML patients were collected and injected into NOD/SCID donor mice to induce AML PDX model. Bone marrow (BM) hematopoietic stem cells were isolated from AML donor mice and then infected with lentiviruses expressing GFP-tagged wild-type hCINAP or hCINAP shRNA. These two types of AML-BM hematopoietic stem cells were then injected into the tail vein of recipient mice to induce AML mice with depleted hCINAP or wild-type hCINAP (scrambled control), respectively. To mimic the clinical medication practice, at D10 after injection, we administered the 7 + 3 chemotherapy to the mice (Fig. 8a) and investigated the effects of hCINAP depletion on cell survival, apoptosis, spleen morphology, and mice survival in response to the chemotherapy.

At D20 after injection, PB cells were collected to detect cell apoptosis by TdT-mediated dUTP Nick-End Labeling (TUNEL) fluorescence assay. In the AML mice, hCINAP knockdown resulted in increased cell death (TUNEL^+^) (Fig. 8b). At D26, two mice in each group were euthanized and the spleen samples were collected for immunohistochemistry to investigate the expression of γ-H2AX and Ki67 (Fig. 8c). The mice induced with knocked down hCINAP AML showed decreased cell proliferation (Ki67^+^) and increased γ-H2AX. These results indicate that hCINAP depletion results in higher levels of DNA damage and renders cells more sensitive to chemotherapeutic drugs. Two mice in each group were sacrificed and the femur BM cells were isolated at D26 and subjected to fluorescence-activated cell sorting (FACS) to analyze the proliferation of xenografted GFP-tagged BM cells. Malignant BM cell proliferation and metastases decreased in hCINAP knockdown mice (Fig. 8d). At the end of the experiment, the spleens of AML mice were photographed and weighed (Fig. 8e, f). In comparison with the hCINAP shRNA AML mice, larger and heavier spleens in the control shRNA group revealed that the histology of hCINAP wild-type AML mice resembles the advanced tumor stages. (Fig. 8e, f). The Kaplan–Meier analysis of the two groups of AML mice revealed that the AML mice with hCINAP knockdown had significantly longer survival (Fig. 8g).

We investigated the effects of hCINAP knockdown on KG-1α cell survival in response to IR and the representative AML therapeutic agents, DNR and HD-Ara-C. Soft agar assays showed that knockdown of hCINAP in AML KG-1α suspended cells resulted in a significant decrease in cell survival rate, and exposing cells to an increased dosage of IR severely aggravated this phenotype (Supplementary Fig. 8a–c). We next performed the apoptosis proportion analyses by using the three samples (healthy control 13, AML 10, and AML 11) mentioned in Fig. 1i. The results showed that healthy control 13 possessed the highest level of hCINAP, with the lowest apoptosis rate, whereas the highest apoptosis rate was observed in AML sample 11 (Fig. 8h). Of note, the level of DSB breaks was elevated by a relatively low dose (4 Gy) IR treatment, indicating that the necrotic white cells from the AML samples not only possessed lower genomic stability but were also more sensitive to IR radiotherapy. The same experiment was performed in the AML KG-1α cell line. After IR treatment, low hCINAP expression in KG-1α resulted in an increased apoptosis rate and more sensitivity to DDR stimuli (Supplementary Fig. 1g). Consistent with this observation, the Kaplan–Meier analysis of overall survival of patients with AML also indicated that the superior curative overall survival ratio was negatively correlated with hCINAP abundance (Fig. 8i).

Altogether, our results indicate that depletion of hCINAP combined with chemotherapy increases DNA-damage, genomic instability, and drug sensitivity in AML PDX mice and AML patient PB cells, promoting regression of AML. Moreover, we examined NPM1 SUMOylation, NPM1-SENP3 interaction, and BRCA1 foci in PB cells and spleen sections from the AML PDX mice. In agreement with our aforementioned molecular mechanism, the NPM1 SUMOylation level is higher in AML mice PB cells and spleen specimens with depleted hCINAP than that of the control group (Fig. 8j, k, Supplementary Fig. 9a). Meanwhile, using spleen sections, PLA staining revealed that knockdown of hCINAP attenuated the interaction between NPM1 and SENP3 (Fig. 8k, Supplementary Fig. 9a); and the immunofluorescence assay showed that the number of BRCA1 foci positively correlated with the levels of SUMOylated NPM1 (Fig. 8k, l and Supplementary Fig. 9b). These data support our model that hCINAP regulates NPM1 SUMOylation and affects BRCA1 protein recruitment, thereby enhances radiotherapy and chemotherapy resistance.

## Discussion

The DDR is a complex system that enables cells to survive and maintain genomic integrity. This process is fine-tuned by many types of posttranslational protein modifications^33^. In this study, we propose a spatiotemporal working model in which NPM1 is SUMOylated in response to DSBs, which is beneficial for BRCA1 recruitment. Meanwhile, hCINAP translocates from the cytosol to the nucleus, where it is recruited to the damaged sites and promotes SENP3-dependent deSUMOylation of NPM1. DeSUMOylation of NPM1 causes dissociation of RAP80 from the damage site and facilitates DNA resection and homologous recombination (Fig. 8m).

Here, we demonstrate that NPM1 SUMOylation responds to IR-induced DSB and is beneficial for maintaining chromosome morphology. The NPM1-depleted AML KG-1α cells that were reintroduced with an NPM1 K263R mutant showed more chromosome breaks than that of KG-1α cells rescued with wild-type NPM1 (Supplementary Fig. 10a). NPM1 SUMOylation is required for the recruitment of DNA repair proteins. Immediately after DNA-damage occurs, NPM1 accumulates at the damage sites and promotes repair. Knockdown of NPM1 leads to a significant decrease in cell survival. Interestingly, we showed that SUMOylation of NPM1 does not affect recruitment of itself to the DSBs (Supplementary Fig. 10b) and the regulatory function of NPM1 SUMOylation in the DDR is independent of NPM1 phosphorylation. These observations suggest that SUMOylation and phosphorylation of NPM1 work independently in response to different types of stimuli and that NPM1 SUMOylation is a responsive regulator of DSBs required for DNA repair and cell survival.

Notably, we identified hCINAP as a negative regulator of NPM1 SUMOylation, which functions at different time points from that of NPM1 in the DDR process. At the later stage of the DDR, hCINAP is recruited to the damage sites and prevents excessive repair by downregulation of NPM1 SUMOylation. Although deletion of hCINAP has no effect on the recruitment of NPM1 to the damage sites (Supplementary Fig. 10c), hCINAP reduces the level of NPM1 SUMOylation and recruitment of repair proteins by promoting the interaction between SENP3 and NPM1. Thus, hCINAP has a critical role in promoting genomic stability.

The role of hCINAP has largely been attributed to its “oncogene” function^16,20,21^, as it is upregulated in solid tumors and correlated with a poor prognosis. However, the functions of hCINAP in hematologic malignancies remained unclear. In this study, we found that excessively low hCINAP protein expression was detrimental in cases of AML. It is notable that hCINAP expression levels are lower in samples collected from AML patients relative to the control group. According to the Oncomine AML database, those patients with AML demonstrating lower hCINAP expression levels show a better prognosis. These results are consistent with the survival analysis of our AML PDX mice model, supporting the hypothesis that lower expression of hCINAP in AML may confer higher error-prone NHEJ repair efficiency and lower error-free HR repair efficiency, to increase the sensitivity to DNR chemotherapy or radiotherapy. The conventional AML therapy is to kill all the leukocytes and then re-establish the blood circulation system by hematopoietic stem cell transplantation. In line with this idea, high levels of drug sensitivity and genomic instability have been associated with improved prognosis. Our results (Fig. 8) reveal that AML cells with depleted hCINAP show higher sensitivity to chemotherapeutics, increased rates of DNA damage and cell death, and alleviated AML progression. Finally, considering the low expression of hCINAP in patients with AML, a combination of an hCINAP regulator and chemotherapy may provide a beneficial approach toward more-efficient AML therapies.

It is worth mentioning that after treatment with different concentrations of DNR or Ara-C for 24 h, the abundance of hCINAP in the leukocytes isolated from PB of AML patients and healthy people changed in distinct patterns. The hCINAP level increased in AML patients, but not healthy people, after drug treatment (Supplementary Fig. 11a, b); yet the level of increased hCINAP was still well below that of the healthy people. As hCINAP is important for maintaining genome integrity, AML cells with low abundance of hCINAP are more sensitive to the drug treatment and thus undergo apoptosis. On the other hand, cells might increase chemotherapy resistance by promoting hCINAP expression. Meanwhile, the different response of hCINAP to the chemotherapy reagents in normal and AML cells suggest that hCINAP is a potential therapeutic target.

The discovery of SUMOylation/deSUMOylation pathways that function to control DNA-damage repair highlights the possibility of modulating these PTM activities to protect healthy cells from the effects of genotoxic anticancer therapies, while till eliminating the cancer cells. As drugs can easily target the ubiquitin-like modification system, such pleiotropic mechanisms can be of substantial use in cancer treatments, offering a number of possibilities for future applications.

## Methods

### Cell lines and clinical samples

HEK293T (CRL-11268), HeLa (CCL-2), and U2OS (HTB-96) cells (purchased from ATCC, USA) were cultured in Dulbecco's Modified Eagle Medium medium (Gibco, USA). KG-1α (CCL-246.1) cells (purchased from ATCC, USA) were cultured in Iscove's Modified Dulbecco's medium (Gibco, USA). OCI-AML2 and OCI-AML3 cells were obtained from Peking University People’s Hospital. All cell lines were tested by PCR to make sure there was no mycoplasma contamination.

All knockout cell lines used in this study were generated using the CRISPR-Cas9 gene editing approach. For CRISPR/Cas9 knockout of human SENP3 in HEK293T and U2OS cells, the following two sgRNAs were used: sgSENP3-1: 5′-CGACTCAAGTCAGGTGGAGGG-3′; sgSENP3-2: 5′-CGAGCCATGAGAGCCTCCGG-3′. For CRISPR/Cas9 knockout of human hCINAP in HEK293T and U2OS cells, the following two sgRNAs were used: sghCINAP-1: 5′-CAGGTACACCAGGGGTTGG-3′; sghCINAP-2: 5′-AGTGGAGTGTTAGTGCTGG-3′. The sgRNA sequences were cloned into the U6-Cas9 plasmid (a gift from Wensheng Wei at Peking University). The sgRNA/Cas9 expression constructs were transiently transfected into HeLa, U2OS, or HEK293T cell lines. Twelve hours after transfection, cells were filtrated via FACS and plated to acquire individual clones. Gene knockout cells were validated and confirmed by immunoblotting and sequencing.

The PB samples were collected the first time when the patients were diagnosed with AML at the Peking University People’s Hospital according to the guidelines of the ethics committees. The PB samples of healthy patients without any sign of hematological malignancies were collected at Peking University Hospital. Informed consent was obtained from all the patients. The investigation was performed under approval by the Ethics Committees of Peking University and Peking University People’s Hospital. Total samples of AML patients included six females and seven males aged from 24 to 80. Total samples of the control group included eight females and five males aged from 19 to 78. White blood cells were separated from the PB using Ficoll-Paque PLUS (GE, USA) and Red Blood Cell Lysis Buffer (Tiangen, China).

### Pharmacological inhibitors, reagents, plasmids, and antibodies

Daunorubicin-hydrochloride (DNR) and HA-Cytarabine (HA-Ara-C) were purchased from Pharmabiology (USA). Duolink In Situ Red Starter Kit Mouse/Rabbit kit used for detecting proximity ligation was purchased from Sigma (USA). SUMOylation Assay Kit was purchased from Abcam (USA). Protease Inhibitor Cocktail was purchased from TOPSCIENCE, TargetMol (USA), Catalog No.C0001.

The expression plasmids pRK-Flag-hCINAP and pRK-HA-NPM1 full-length as well as their corresponding enzymatic mutations and deletion mutants were constructed by inserting hCINAP or NPM1 into the pRK vector at the EcoRI and XbaI restriction sites and verified by DNA sequencing. SENP3 was inserted into the 3Myc-pcDNA vector. His-SUMO3 were inserted into the pEF1-HisB vector.

Antibodies, including anti-Myc (M047-3, WB: 1:1000), anti-GST (M071-3, WB: 1:1000), anti-β-actin (PM053, WB: 1:2000), anti-Lamin B1 (PM064, WB: 1:2000), anti-His (D291-3, WB: 1:1000), and anti-α-Tubulin (M175-3, WB: 1:2000), were purchased from MBL (USA). Monoclonal anti-Flag (M2; F3165, WB: 1:10000; IF: 1000) and anti-HA (HA-7; H9658, WB: 1:10000) were purchased from Sigma (USA). Phospho-Histone H2A.X (Ser139, 059636, WB: 1:5000; IF: 1:100) was purchased from Merck Millipore (USA). DNA-PKcs (sc-5282, WB: 1:1000; IF: 1:100), RNF8 (sc-271462, IF: 1:50), RNF168 (sc-101125, IF: 1:100), RAD51 (sc-53428, sc-133089; IF: 1:50), 53BP1 (sc-22760, IF: 1:100) and BRCA1 (sc-135731, IF: 1:100) were purchased from Santa Cruz. Phospho-DNA-PKcs (Ser2056, WB: 1:1000; IF: 1:100) (ab18192) was purchased from Abcam. Anti-RAP80 (A7244, WB: 1:1000; IF: 1:100), CtIP (A10201, IF: 1:100) and Ki67 (A11390, WB: 1:1000; IHC: 1:100) were purchased from Abclonal (China). Anti-Chk1 (YT0904, WB: 1:1000) was purchased from Immunoway (USA). Anti-NPM1 (#3542, WB: 1:1000; IF: 1:100), anti-Phospho-Chk1 (Ser317, #12302, WB: 1:1000) and anti-SENP3 (D20A10, WB: 1:1000; IF: 1:100) were purchased from Cell Signaling Technology. IRDye 800CW goat anti-mouse (926–32210, 1:1000) and IRDye 800CW goat anti-rabbit (926–32211, 1:1000) were purchased from LI-COR Bioscience (USA). Fluorescein isothiocyanate-conjugated goat anti-mouse IgG (ZF-0312, 1:1000) and TRITC-conjugated anti-rabbit IgG (ZF-0316, 1:1000) were purchased from ZSGB-Bio (China). Rabbit polyclonal anti-hCINAP was generated by immunizing a rabbit with the purified hCINAP protein.

### Immunofluorescence

The recruitment of repair proteins to the damage site (BRCA1, RAP80, CtIP, RAD51, 53BP1, and the phosphorylation of 53BP1) under different IR treatment lengths was investigated in U2OS cells and PB leukocytes from patients with AML. U2OS cells were treated with or without IR at 10 Gy and collected after resting for 1 h or 12 h. AML patient leukocyte samples were adhered to glass slides using a Thermo Scientific Cytospin 4 Cytocentrifuge. Images were visualized with a confocal laser-scanning microscope (Zeiss LSM-710 NLO and DuoScan, Germany) using a × 63 oil objective lens. The number and intensity of IR-induced nuclear foci were quantified using the Imaris 7.6 software (Bitplane, UK). The DNA-damage marker, γ-H2AX, was used to indicate the lesion points. Nuclear DNA was stained with 4′,6-diamidino-2-phenylindole (DAPI; 1 g/mL).

### Histology and immunohistochemistry

Spleens excised from mice were fixed in 10% paraformaldehyde overnight and embedded in paraffin. The maximum cross sections (3 μm thick) were picked; primary antibodies were used for the following staining: hCINAP, SUMO2/3, Ki67, and γ-H2AX. HRP-conjugated secondary antibodies were used, and the signal was visualized using DAB (3, 3-diaminobenzidine). Apoptosis was detected by TUNEL using the In situ Cell Death Detection Kit, Fluorescein following the manufacturer’s instructions (Beyotime, China).

### Laser-induced DNA-damage and live cell imaging

Laser micro-irradiation is a specialized method to observe DSB protein recruitment. U2OS cells were grown in a glass-bottomed petri dish and topically irradiated with a 365-nm pulsed nitrogen laser (16 Hz pulse, 41% laser output) generated from a micropoint system (Andor). This system was directly coupled to the epifluorescence path of the Nikon A1 confocal imaging system, and time-lapse images were captured every 10 s for the indicated time. The signal intensity of the irradiation path from > 20 cells was calculated using the ImageJ software.

### Metaphase spread preparation

Cells were treated with 10 ng/ml Nocodazole (Sigma) for 12 h and fixed (ice-cold 75% methanol). After three washes in fixative solution, cells were spread on glass slides, steam treated for 5 s, heat dried, and stained with mounting media containing DAPI. Spreads were imaged using a Nikon E1000 epifluorescence microscope. At least 30 spreads were analyzed in each experimental group.

### In vitro SUMOylation assay

For in vitro SUMOylation, GST-tagged NPM1 protein was purified from *Escherichia coli* and diluted in SUMOylation buffer, immunoprecipitated with Mg-ATP, SUMO E1, SUMO E2, and SUMO2/3 compound. The mixture was incubated for 1 h, following the manufacturer’s manual (Abcam). The SUMOylated GST-NPM1-SUMO2/3 was then analyzed by GST pull down.

### Proximity ligation assay

Proximity ligation assay was performed with the DuoLink kits (Sigma-Aldrich). Cells expressing indicated proteins were fixed with 4% paraformaldehyde. Then, the cells were permeabilized with 0.5% Triton X-100 and incubated with commercial blocking solution. Mouse and rabbit species antibodies were mixed and diluted in the antibody diluent. The PLUS and MINUS PLA probes were mixed and diluted in antibody diluent to incubate for 1 h. After incubated with ligase and washed with commercial Wash Buffer, the samples were mounted with Prolong Diamond Antifade Mountant with DAPI. Images were recorded using a Zeiss LSM-710 confocal microscope system with a × 63 lens and were analyzed with Zeiss confocal software. The quantification was done using ImageJ software.

### Neutral comet assay

Neutral comet assay was performed to detect DSB-induced genomic instability^34^. Cells subjected to radiation (8 Gy, 1 h, or 12 h recovery) were harvested and resuspended in ice-cold phosphate-buffered saline (PBS). The neutral single-cell agarose gel was put in the electrophoresis running buffer to allow for DNA helix uncoiling. The amount of damaged DNA was visualized by single-cell agarose gel electrophoresis under alkaline conditions. Observations were performed under an epifluorescence microscope (Olympus, Japan). Quantitation was performed on 60–100 nuclei from each gel using Comet Assay IV software (Perceptive Instruments, UK). Olive tail moment was used as the parameter of this assay instead of tail length or tail DNA content, since it is independent of the comet shape and can better represent DNA-damage status.

### DNA repair assays

In NHEJ assays, *hCINAP*^*WT*^ and *hCINAP*^−/−^ cells were transfected with linearized pcDNA3.1/puromycin (Invitrogen) and the pEG FP-C1 plasmid. After 36 h, the cells were collected, counted, and plated on two plates. The transfection efficiency was determined and normalized to EGFP expression. In HR assays, *hCINAP*^*WT*^ and *hCINAP*^−/−^ cells were co-transfected with DR-GFP, an I-SceI expression vector, and a DsRed plasmid. Cells were harvested and washed with 1 × PBS 48 h after transfection. Green (EGFP) and red (DsRed) fluorescence were measured by FACS on a FACSVerse instrument (BD Biosciences, USA). The ratio of EGFP and DsRed double-positive cells to DsRed positive cells was taken as the repair efficiency. The results were normalized to those of *hCINAP*^*WT*^ cells. Samples were analyzed using FlowJo software to determine GFP-positive cells relative to cells expressing DsRed. U2OS-DR-GFP cells that were only transfected with DsRed but lacked I-SceI were considered as the negative control (background level of HR). Repair frequencies were normalized to those of *hCINAP*^*WT*^.

### PDXs AML mice

All animal experiments were approved by the Peking University Laboratory Animal Center. The donor male NOD/SCID mice (6–8 weeks old) were purchased from Beijing Vital River Laboratory Animal Technology and housed at Peking University Laboratory Animal Center following the ‘Principles for the Utilization and Care of Vertebrate Animals’ and “Guide for the Care and Use of Laboratory Animals”. The serial number of production license for laboratory animals (SCXK) is SCXK-2016-0010. The serial number of use license for laboratory animals (SYXK) is SYXK-2016-0028. BM cells were isolated from the donor mice. In vitro proliferation and induction of the BM cells were performed, followed by GFP-tagged lentiviral infection to get two types of AML-BM hematopoietic stem cells, the CINAP knockdown and the control group. The two cell types were transplanted intravenously into 28 recipient mice (14 per group, 10^5^ cells transplanted in each mouse) at Day0 (D0). In the next 10 days, leukemia development was monitored daily by physical appearance. To mimic the clinical medication practice, “7 + 3” chemotherapy (DNR from D10 to D12, HA-Ara-C from D10 to D16) was administered by tail vein injection. PB was analyzed by FACS to assess leukemia development at D20. The leukemia load (human CD^45+^ cells) in the caudal vein, BM, and spleen were determined by TUNEL, FACS, and immunohistochemistry analyses at D26. The death date of every mouse in the two groups was recorded and the survival rate of each group was calculated at the end.

### Bioinformatics analysis

RNA-seq analysis was performed by ANNOROAD. In brief, the total RNA from wild-type and knockout hCINAP U2OS cells was extracted, and sequencing was performed with Illumina Solexa Ultrasequencing. The downstream analysis includes differential expression analysis and KEGG pathway enrichment. For analyzing the expression level of hCINAP in patients with AML, the recomputed gene expression data sets of TCGA LAML cancer type and GTEx BM that were based on the gencode v23 gene model from UCSC Xena (http://xena.ucsc.edu/) were downloaded. The clinical data of TCGA LAML cancer type were downloaded from the GDC Data Portal (https://gdc-portal.nci.nih.gov/). The expression unit was TPM (transcript per million). We compiled the data sets of TCGA LAML cancer and GTEx BM tissue for differential expression and survival analyses. The sample set included 243 samples (including survival information of 173 patients and 70 normal samples).

### Statistical analysis

The statistical results were obtained from at least three independent biological replicates. Detailed *n* values for each panel in the Figs. are stated in the corresponding legends. All results were presented as mean ± SEM unless otherwise stated. *P* values were obtained via the Student’s *t* test (two-tailed), Mann–Whitney test (for two group comparisons) or one-way ANOVA using GraphPad Prism 8.0 software. *P < 0.05, **P < 0.01, ***P < 0.001. Survival curves were obtained from Kaplan–Meier estimates and validated with the log-rank test.

### Reporting summary

Further information on research design is available in the Nature Research Reporting Summary linked to this article.

## Supplementary information

Supplementary information

Peer Review

Reporting Summary

Description of Additional Supplementary Files

Supplementary Data 1

## Data Availability

A reporting summary for this Article is available as a Supplementary Information file. Unprocessed scans of western blots are provided in Supplementary Fig. 13. The source data underlying Figs. 1h, 2a, 4a, and Supplementary Fig. 1h are deposited in the figshare (10.6084/m9.figshare.c.4438148.v2). RNA-sequencing data are available under the accession code GSE134342 at Gene Expression Omnibus (GEO). All data are available from the corresponding author upon reasonable request.
