# Supplementary material for: hCINAP regulates the DNA-damage response and mediates the resistance of acute myelocytic leukemia cells to therapy
Source: Nat Commun. 2019 Aug 23;10:3812. doi: 10.1038/s41467-019-11795-5 (PMC6707248; doi:10.1038/s41467-019-11795-5)
Supplement: Supplementary file 1 — Supplementary information [file 41467_2019_11795_MOESM1_ESM.pdf]

Supplementary Information

hCINAP regulates the DNA damage response and mediates the resistance of acute myelocytic leukemia cells to therapy

Xu et al

Supplementary Figure 1

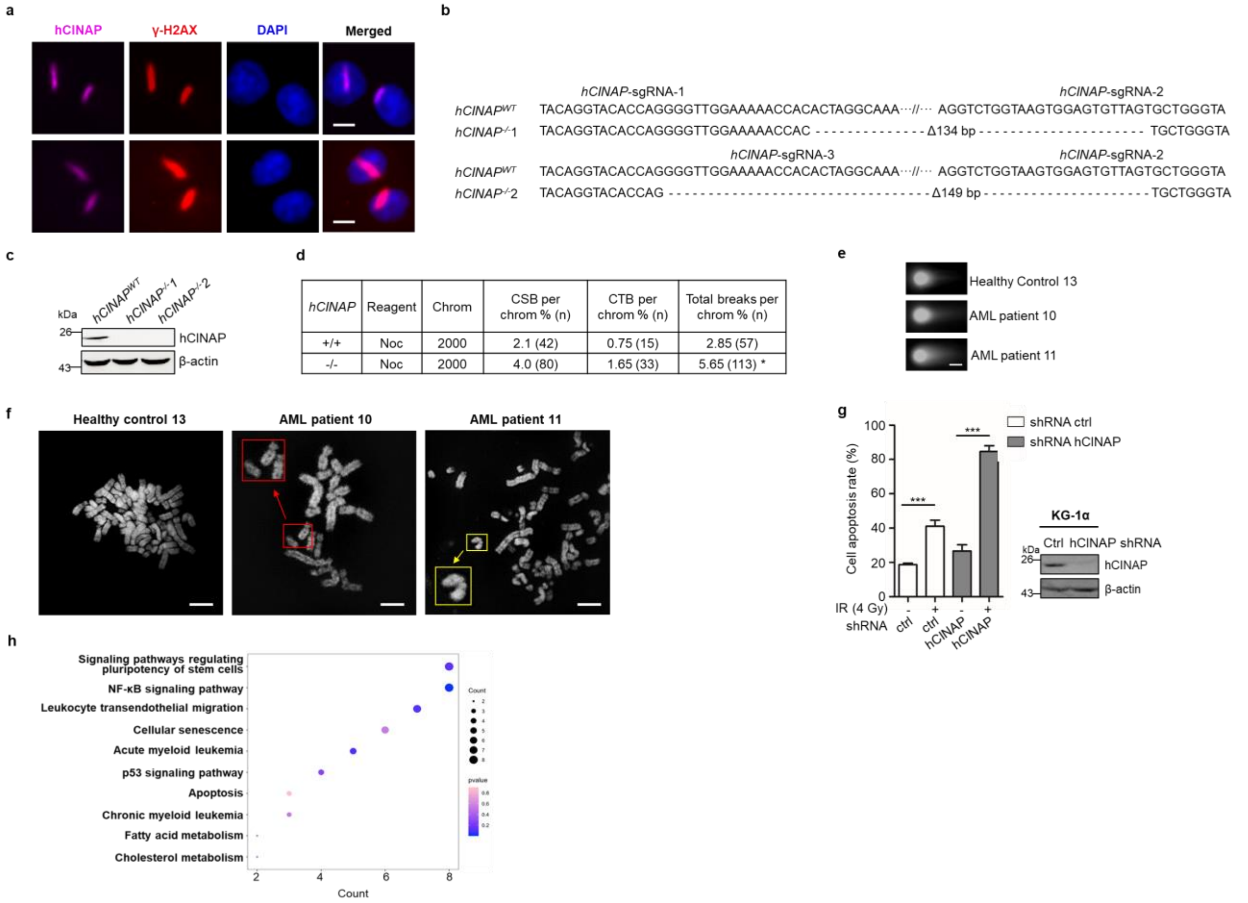

Supplementary Figure 1 | hCINAP is essential for genome stability and is correlated with AML. a Endogenous hCINAP co-localizes with γ-H2AX at DSB sites induced by laser micro-irradiation. U2OS cells were subjected to laser micro-irradiation, after 1 hour remission in 37℃ incubator, cells were immunostained with indicated antibodies. **b** Partial coding sequences of human *hCINAP* exon 1, intron 1, and the respective sequencing results for the mutated alleles of *hCINAP*$^{-/-}$ clone 1 and 2 are shown. **c** Knockout of *hCINAP* was confirmed by western blot using anti-hCINAP antibodies. **d** A total of 2000 chromosomes for each *hCINAP*^{WT} and *hCINAP*$^{-/-}$ cell line were karyotyped and chromosome abnormalities were detected, one-way analysis of variance (ANOVA), **P* < 0.05. **e** Neutral comet assays were performed to assess genomic stability of healthy control 13, AML patient 10 and 11. More than 100 cells were counted in each experimental group. The representative olive tail images were shown. **f** Representative images of chromosome spreads from cells in Figure **1i** are shown. Scale bar, 2 μm. **g** Knockdown of hCINAP in AML KG-1α cells resulted in a significant increase in the IR-treated cell apoptosis rate. KG-1α cells were treated with 4 Gy IR and then performed using Annexin V-FITC apoptosis detection kit. Statistical analysis was

performed using the Student's *t*-test; ***$P < 0.001$. More than 10^5 cells were counted in each experimental group. **h** KEGG pathway enrichment analysis of *hCINAP*^{WT} and *hCINAP*$^{-/-}$ U2OS cells. Pathways are selected according to the relevance to AML and represented in bubble plots. Unprocessed scans of blots are provided in Supplementary Figure 13.

Supplementary Figure 2

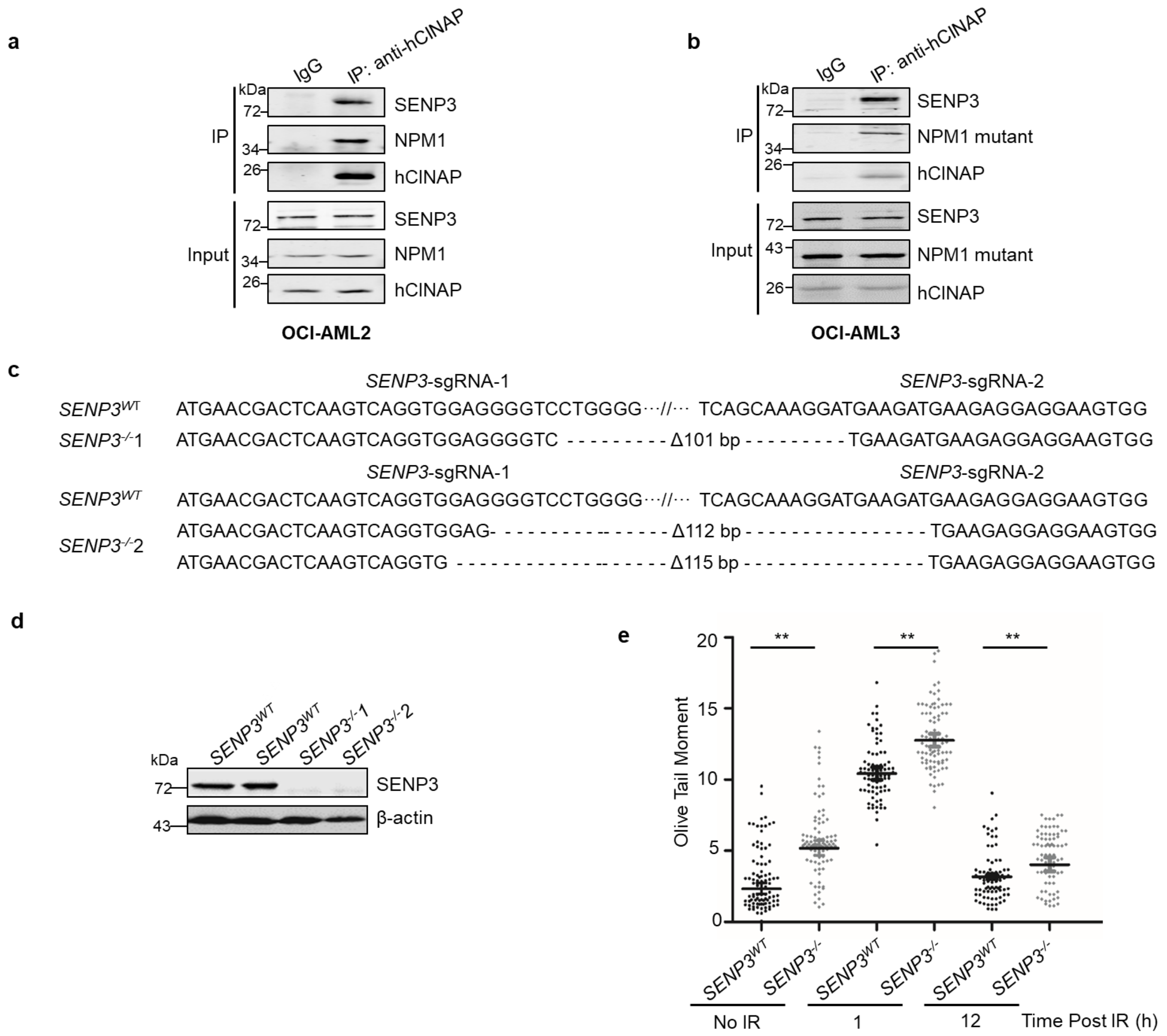

Supplementary Figure 2 | SENP3 is involved in the DNA damage repair. a, **b** The interaction between endogenous hCINAP and NPM1, hCINAP and SENP3 in OCI-AML2 cells, hCINAP and NPM1 mutant, hCINAP and SENP3 in OCI-AML3 cells were confirmed by Co-IP assay using the indicated antibodies. **c** Partial coding sequences of the human *SENP3* exon 1 and the respective sequencing results for the mutated alleles of *SENP3*$^{-/-}$ clone 1 and 2 are shown. **d** Knockout of SENP3 was proved by western blot analysis using anti-SENP3 antibody. **e** *SENP3*^{WT} and *SENP3*$^{-/-}$ HEK293T cells were treated with 10 Gy of irradiation and collected at the indicated time points and then subjected to the neutral comet assay. The olive tail moments were quantified using the Student's *t*-test; $*P < 0.05$, $**P < 0.01$. More than 100 cells were counted in each experimental group. Unprocessed scans of blots are provided in Supplementary Figure 13.

Supplementary Figure 3

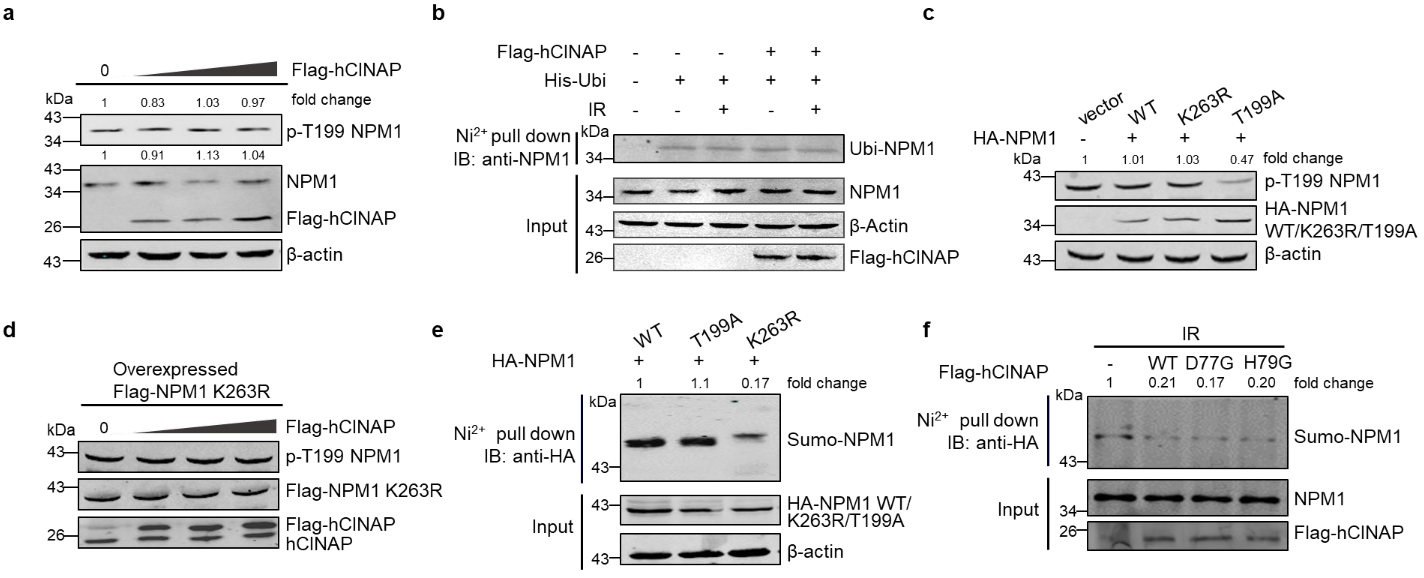

Supplementary Figure 3 | hCINAP does not affect NPM1 ubiquitination and phosphorylation. a hCINAP does not affect the expression or phosphorylation of NPM1. U2OS cells were transfected with different concentrations of Flag-tagged hCINAP. Abundance of the NPM1 protein and its phosphorylation level at Thr199 were examined via immunoblotting using an antibody specifically targeting wild-type and T199-phosphorylated NPM1. **b** hCINAP does not affect NPM1 ubiquitination. HEK293T cells transfected with His-ubiquitin, Flag-empty vector, or Flag-hCINAP were treated with or without IR (6 Gy). The ubiquitination of endogenous NPM1 was examined via His-ubiquitination pull-down assays using an anti-NPM1 antibody. **c-e** NPM1 SUMOylation and phosphorylation seen have no crosstalk. U2OS cells were transfected with the indicated plasmids. The phosphorylation of NPM1 at Thr199 was examined via immunoblotting using specific anti-NPM1 T199-phosphorylated antibodies (**c**, **d**), and the effect of NPM1 phosphorylation on its SUMOylation was detected by his-SUMO pull-down (**e**)**. f** hCINAP regulates NPM1 SUMOylation not depends on its adenylate kinase ability. HEK293T cells transfected with Flag-empty vector, Flag-hCINAP or enzymatic activity-depleted mutants hCINAP-D77G, hCINAP-H79G were treated with or without IR (6 Gy) and released for 1 hour. The SUMOylation of endogenous NPM1 was examined via his-SUMOylation pull-down assays using an anti-NPM1 antibody. Unprocessed scans of blots are provided in Supplementary Figure 13.

Supplementary Figure 4

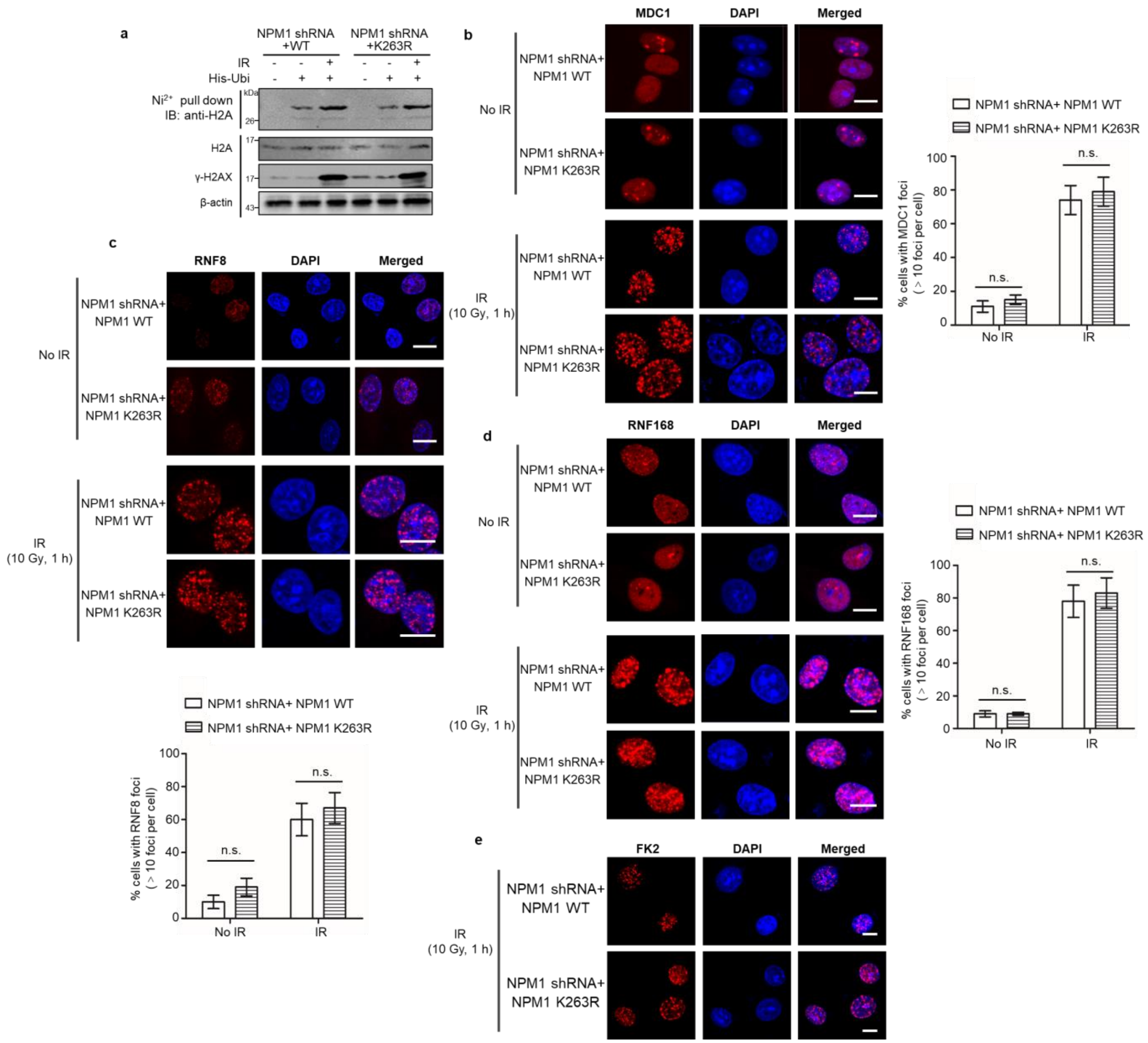

Supplementary Figure 4 | NPM1 SUMOylation does not affect H2A ubiquitination and IR-induced recruitment of MDC1, RNF8, RNF168, and FK2. a NPM1 SUMOylation does not affect H2A ubiquitination. HEK293T cells with depleted NPM1 were transfected with NPM1 WT or K263R mutant. The SUMOylation of endogenous H2A was examined by his-SUMOylation pull-down assays using an anti-H2A antibody. **b-e** U2OS cells infected with lentivirus harboring NPM1 shRNA were transfected with NPM1 WT or K263R mutant, followed by treatment with or without IR (10 Gy), and then subjected to immunofluorescence assays using anti-MDC1 (**b**), RNF8 (**c**), RNF168 (**d**) and FK2 (**e**) antibodies respectively. The immunofluorescence results are presented as the mean ± SEM of three biological

replicates. The statistical analysis was performed using the Student' s *t*-test; $^{**}P < 0.01$, $^{***}P < 0.001$, n.s. is short for not specified. Approximately 100 cells in each group were counted. Scale bar, 10 μm. Unprocessed scans of blots are provided in Supplementary Figure 13.

Supplementary Figure 5

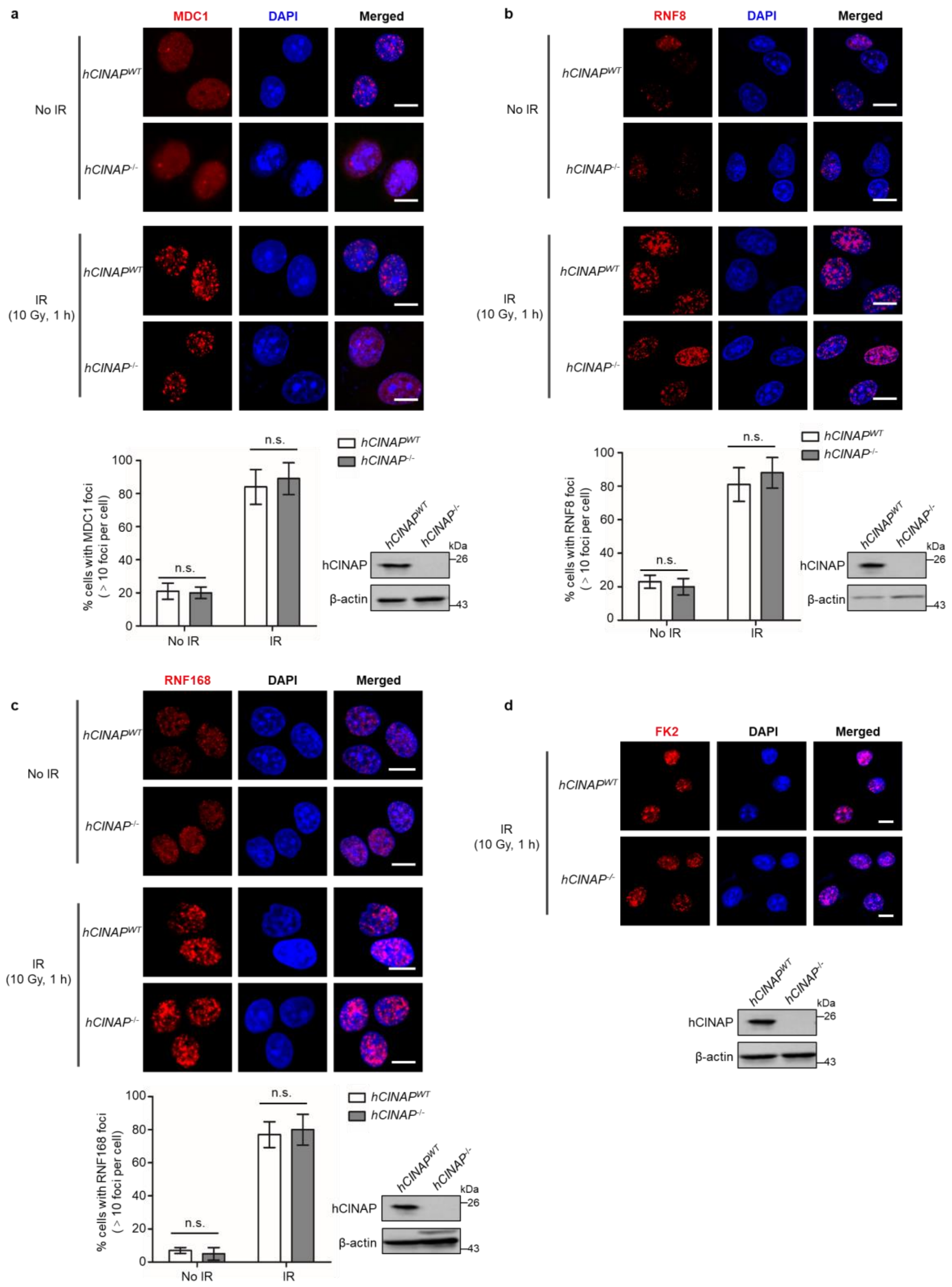

Supplementary Figure 5 | hCINAP does not affect IR-induced recruitment of MDC1, RNF8, RNF168, FK2. a-d *hCINAP*^{WT} and *hCINAP3*$^{-/-}$ HEK293T cells were treated with or without IR (10 Gy) and subjected to immunofluorescence assays using anti-MDC1 (**a**), anti-RNF8 (**b**), anti-RNF168 (**c**) and anti-FK2 (**d**)

antibodies, respectively. The immunofluorescence results are presented as the mean ± SEM of three biological replicates. The statistical analysis was performed using the Student' s *t*-test; $**P < 0.01$, $***P < 0.001$, n.s. is short for not specified. Approximately 100 cells in each group were counted. Scale bar, 10 μm. Unprocessed scans of blots are provided in Supplementary Figure 13.

Supplementary Figure 6

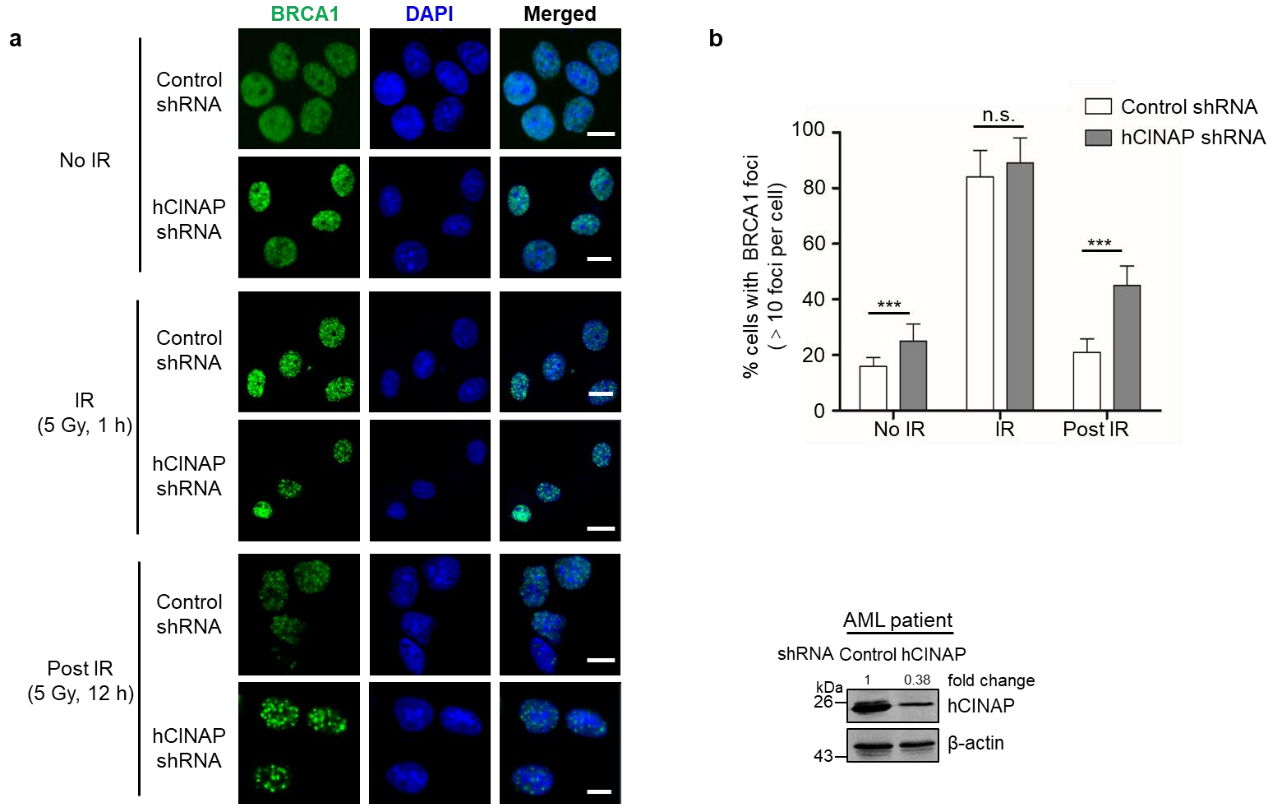

Supplementary Figure 6 | Low hCINAP expression in AML patients show more BRCA1 foci recruitment. a Knockdown of hCINAP expression in AML peripheral blood white cells increases the foci formation of BRCA1 after DNA damage. Cells were treated with or without low dose IR (5 Gy) and subjected to immunofluorescence assays using an anti-BRCA1 antibody. Immunofluorescence images and percentage of cells with more than 10 BRCA1 foci were counted. **b** The immunofluorescence results are presented as the mean ± SEM of three biological replicates. The statistical analysis was performed using the Student's *t*-test; $**P < 0.01$, $***P < 0.001$. Approximately 100 cells in each group were counted. Scale bar, 10 μm. Unprocessed scans of blots are provided in Supplementary Figure 13.

Supplementary Figure 7

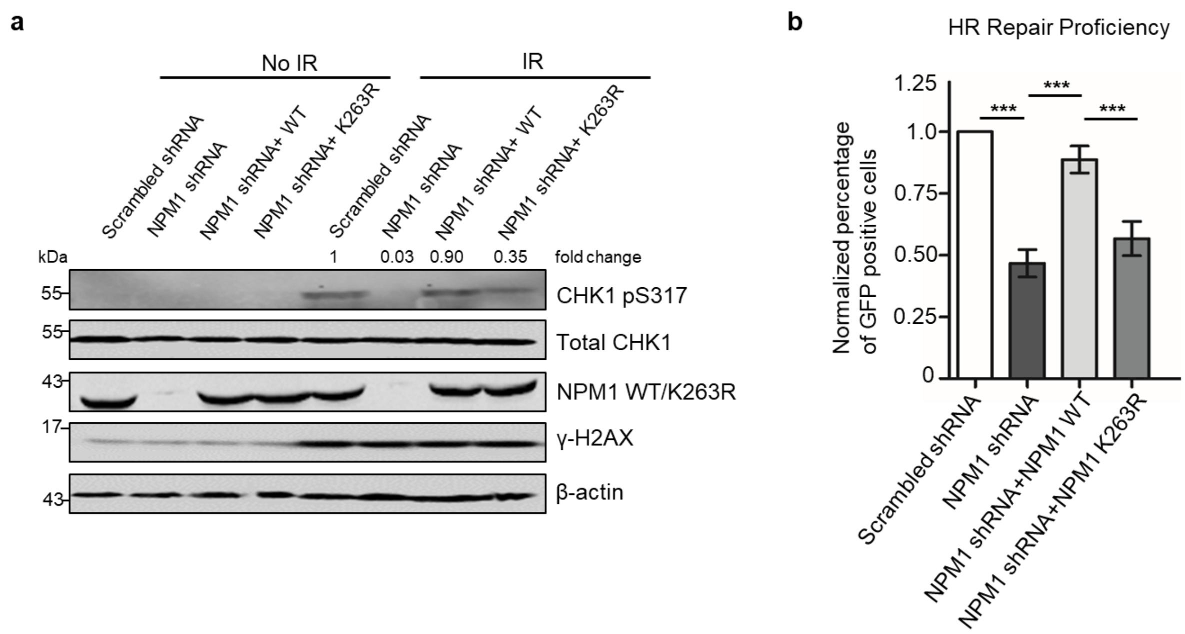

Supplementary Figure 7 | NPM1 SUMOylation is beneficial for HR repair proficiency. a HEK293T cells transfected with indicated plasmids were treated with or without IR (4 Gy) , and subjected to western blot analyses to access the abundances of total CHK1 and P-CHK1 S317 using specific antibodies. **b** U2OS cells were infected with control shRNA or NPM1 shRNA and rescued with NPM1 WT or K263R mutant. At 48 hours afterinfection, cells were transfected with DR-GFP, I-SecI and dsRed vectors for 48 hours, and then the percentage of GFP-positive cells was determined by FACS. Results represent mean ± SEM of three independent experiments. Knockdown efficiency was confirmed by western blotting. Unprocessed scans of blots are provided in Supplementary Figure 13.

Supplementary Figure 8

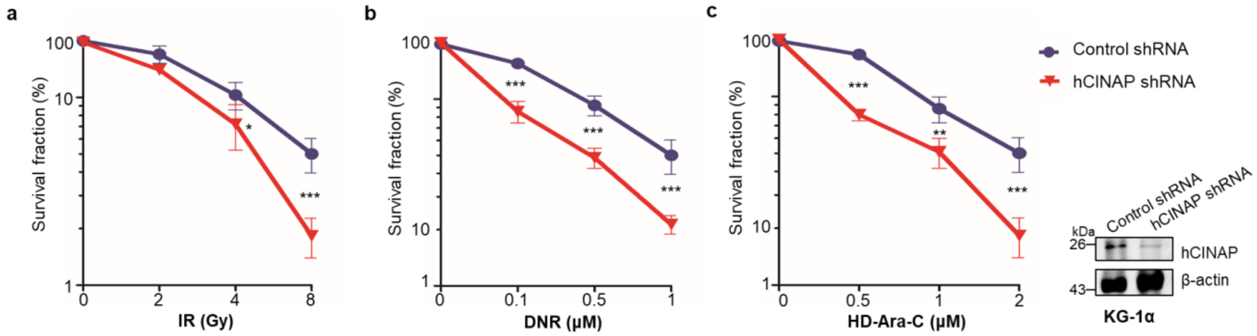

Supplementary Figure 8 | AML cells with depleted hCINAP exhibit increased sensitivity to ionizing radiation and DNA damaging agents. a-c, AML KG-1α cells transfected with hCINAP shRNA or control shRNA were treated with the indicated doses of IR (**a**), DNR (**b**) and HD-Ara-C (**c**), and then subjected to soft agar assays to examine the effects of hCINAP depletion on cell survival. Statistical analysis was performed using the Student's *t*-test; $*P < 0.05$, $**P < 0.01$, $***P < 0.001$. Unprocessed scans of blots are provided in Supplementary Figure 13.

Supplementary Figure 9

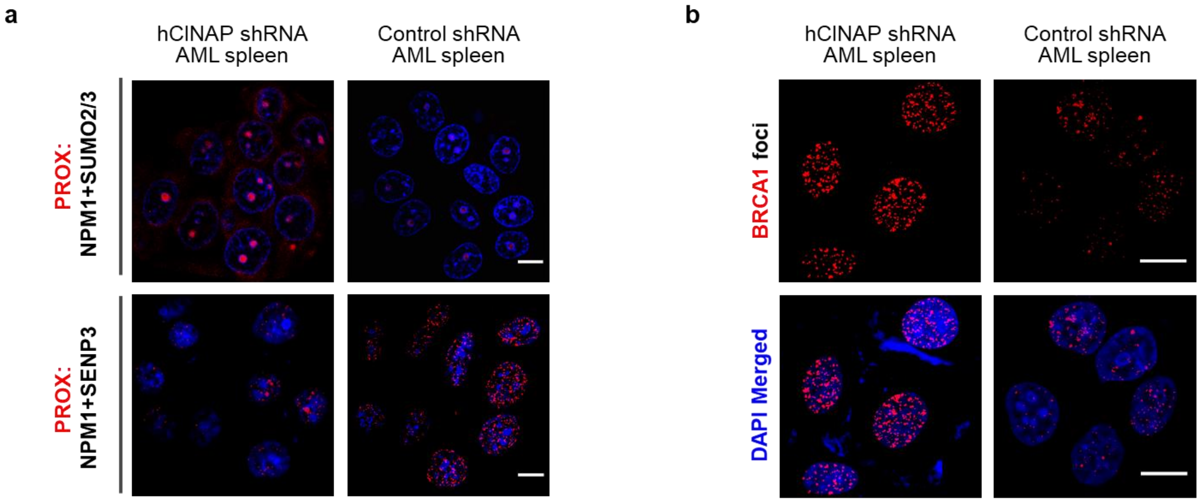

Supplementary Figure 9 | hCINAP deficiency promotes NPM1 SUMOylation and BRCA1 foci in AML mice spleen cells. a Representative pictures of PLA analysis using hCINAP shRNA and Control shRNA AML mice spleens specimens. Scale bar, 10 mm. Upper panel: NPM1-SUMO2/3 PLA. Lower panel: NPM1-SENP3 PLA. **b** Representative images of BRCA1 foci detected by IF analysis with hCINAP shRNA and Control shRNA AML mice spleens specimens. Scale bar, 10 mm.

Supplementary Figure 10

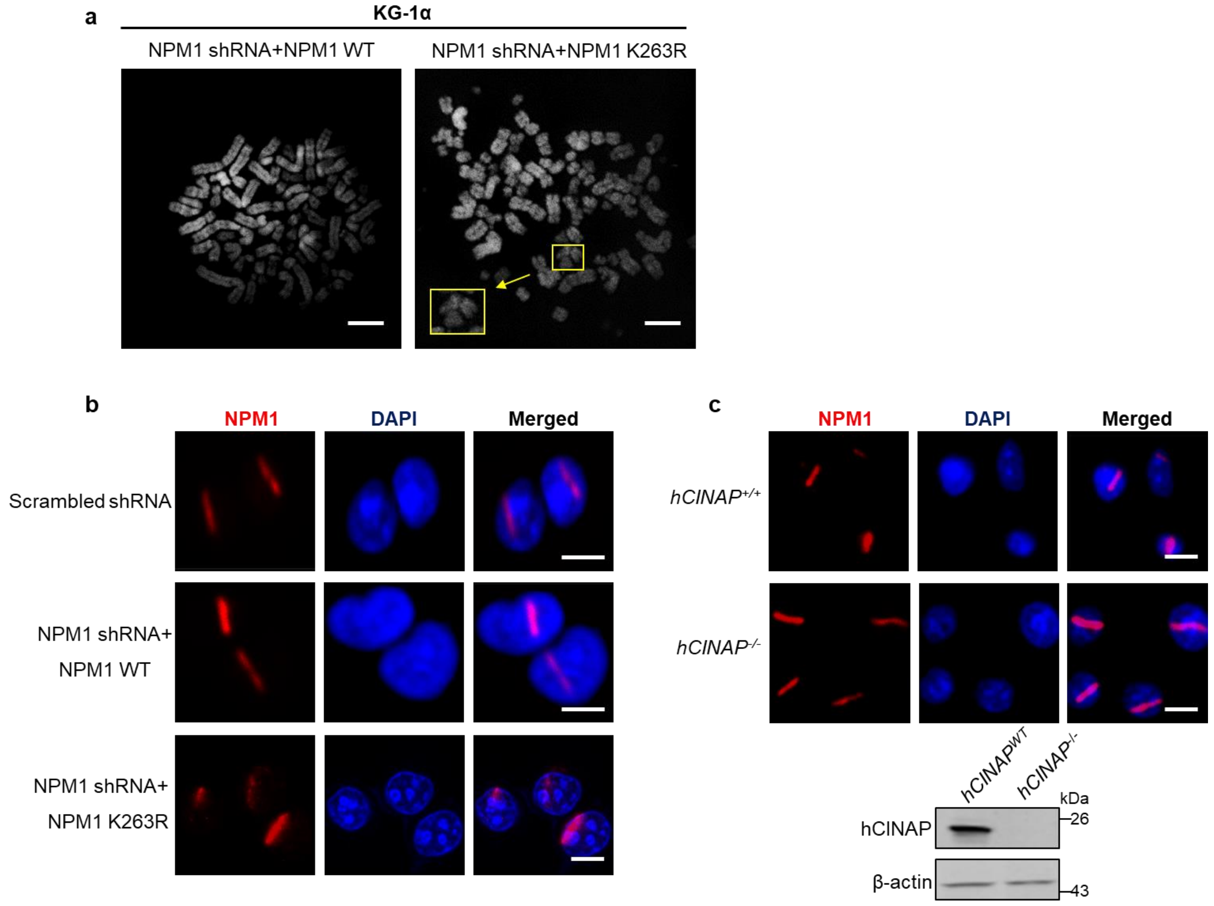

Supplementary Figure 10 | Endogenous NPM1 is recruited to DNA damaged sites and its SUMOylation is benefit for genomic stability. a Representative images of chromosome spreads from NPM1 wild-type and acute myeloid leukemic KG-1α (SUMOylation-deficient mutant) cells are shown. Scale bar, 2 μm. **b**, **c** U2OS cells were subjected to laser micro-irradiation. The laser treated cells were fixed by dried methyl alcohol and done the immunofluorescence assays using anti-NPM1 antibody. Accumulation of NPM1 at the damage sites were detected by fluorescent microscopy. Images are shown, scale bar, 10 μm. Unprocessed scans of blots are provided in Supplementary Figure 13.

Supplementary Figure 11

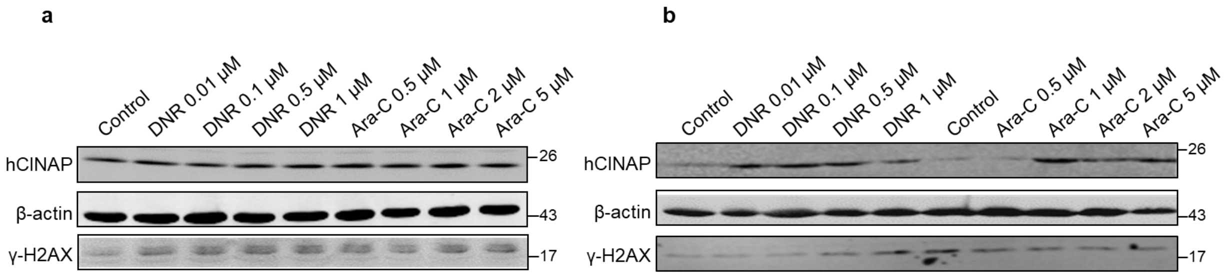

Supplementary Figure 11 | a, **b** The peripheral white blood cells from healthy person (**a**) and AML patients (**b**) were harvested and treated with different concentration of DNR or Ara-C for 48 hours, and the levels of hCINAP were examined by immunoblot using anti-hCINAP antibody. Unprocessed scans of blots are provided in Supplementary Figure 13.

Supplementary Figure 12

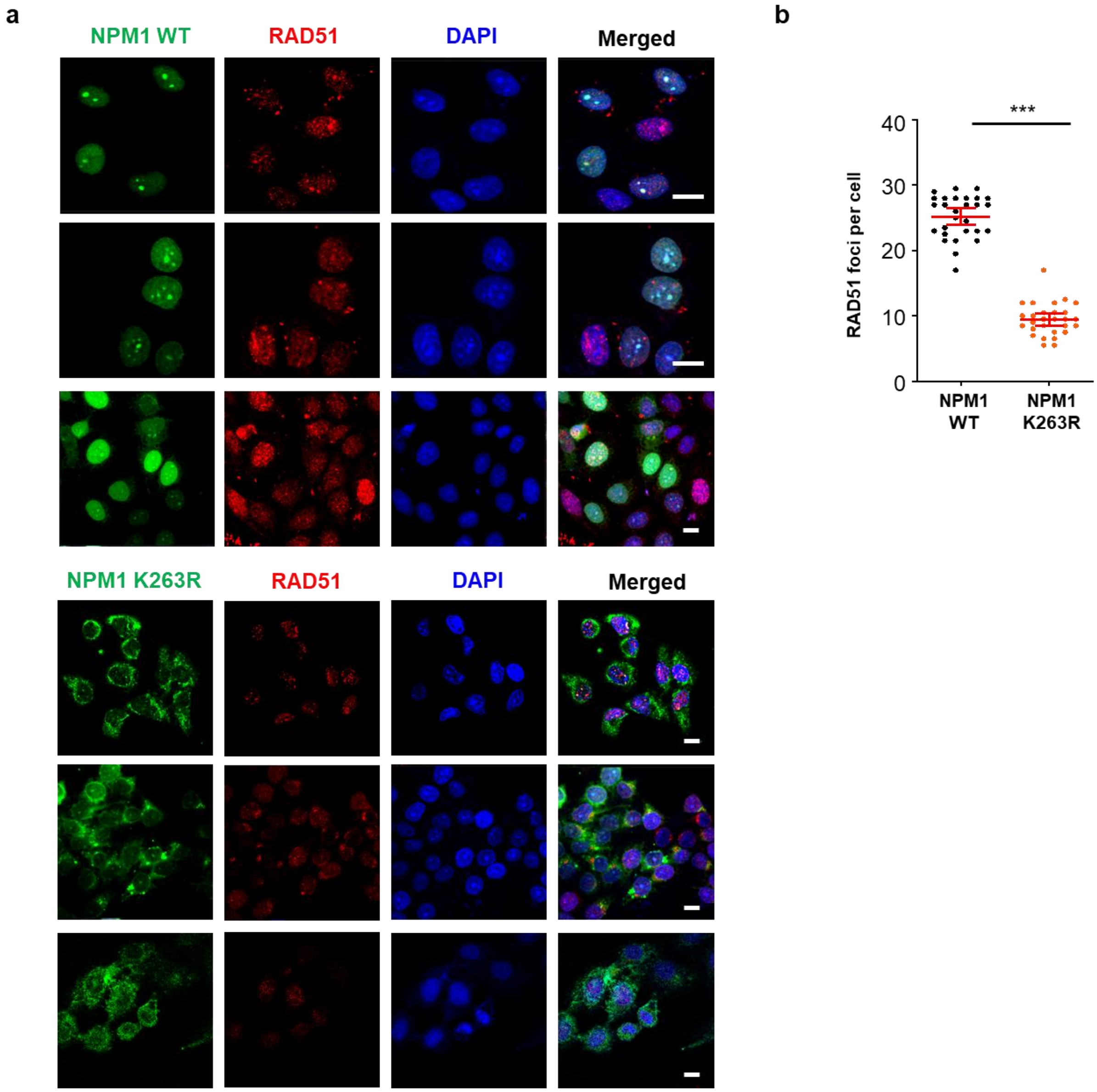

Supplementary Figure 12 | a, **b** The immunofluorescence data in **Figure 5k** was strengthened by providing additional cell images. (**a**) Representative pictures of combinatorial immunofluorescence staining for RAD51 and NPM1 WT/ K263R are shown, as indicated. DAPI was used to visualize cell nuclei. The scale bars represent 10 μm. (**b**) Quantification of RAD51 foci in each group were counted. The results were presented as the mean ± SEM. Statistical analysis was performed using the Student's *t*-test; $^{***}P < 0.001$. Approximately 50 cells in each group were counted.

Supplementary Figure 13

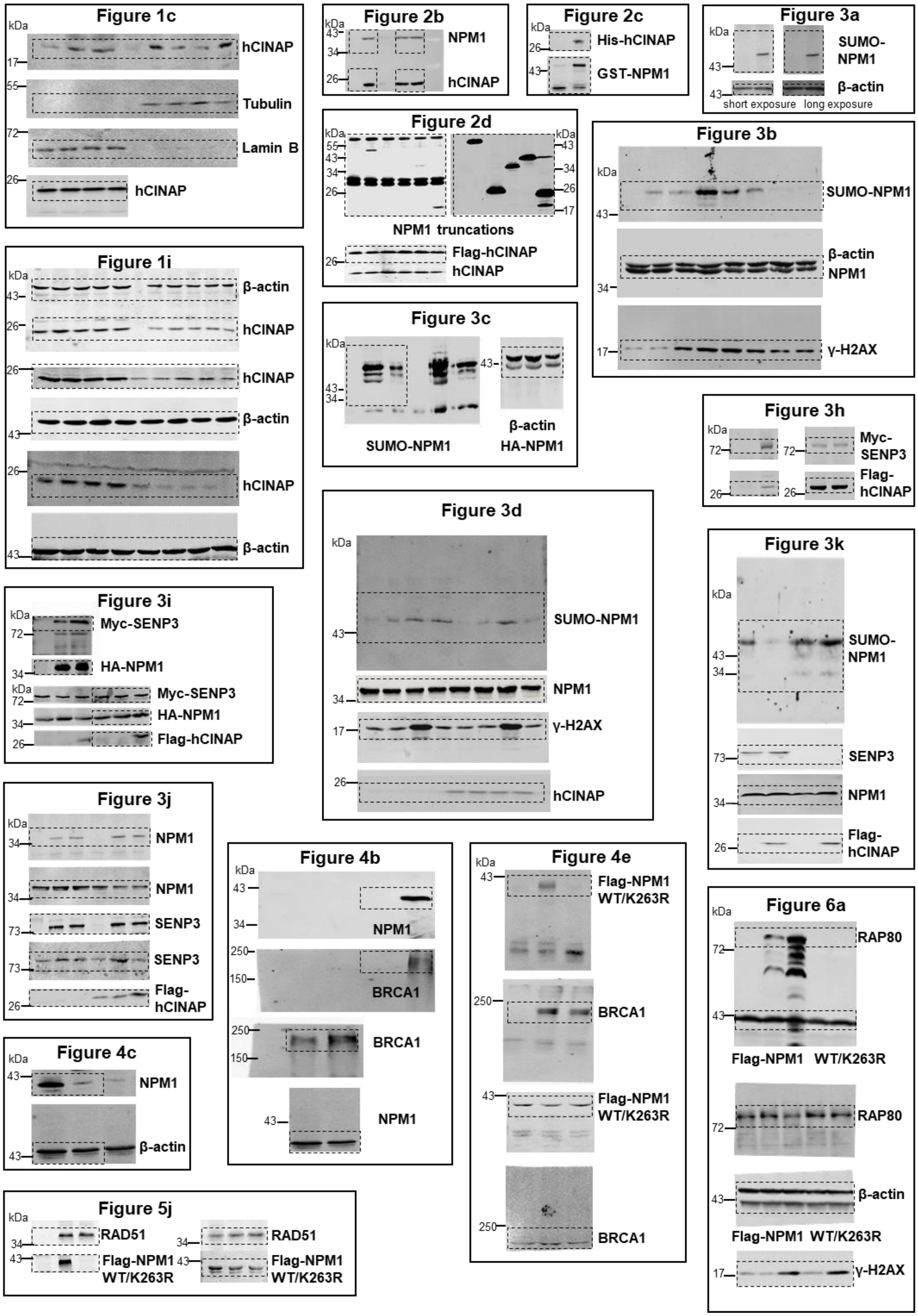

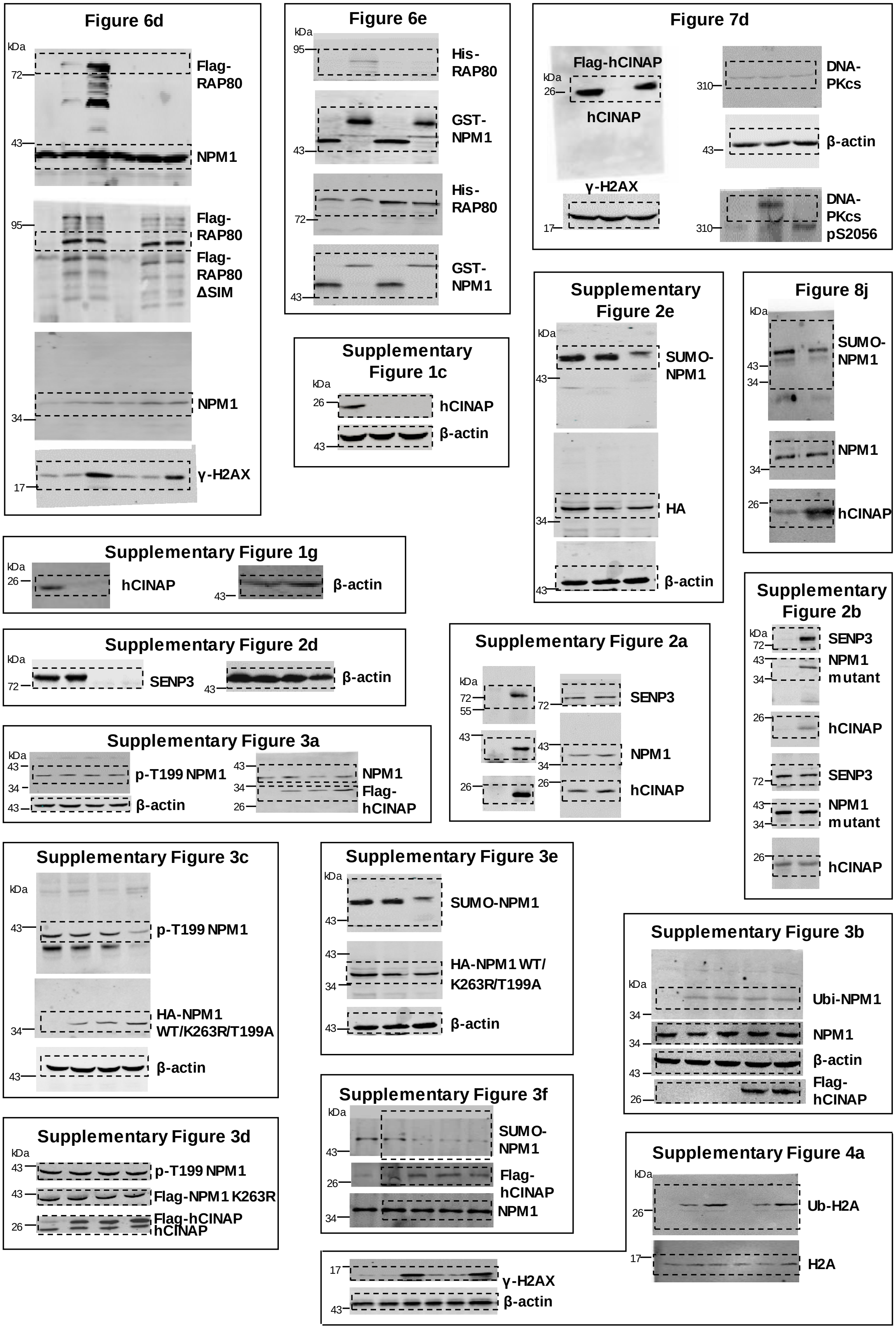

Figure 6d
kDa
72
43
95
34
17
Flag-RAP80
NPM1
Flag-RAP80
Flag-RAP80 ΔSIM
NPM1
γ-H2AX
Figure 6e
kDa
95
43
72
His-RAP80
GST-NPM1
His-RAP80
GST-NPM1
Figure 7d
Flag-hCINAP
kDa
26
hCINAP
γ-H2AX
17
310
43
DNA-PKcs
β-actin
DNA-PKcs pS2056
Supplementary Figure 1c
kDa
26
43
hCINAP
β-actin
Supplementary Figure 2e
kDa
43
34
SUMO-NPM1
HA
β-actin
Figure 8j
kDa
43
34
26
SUMO-NPM1
NPM1
hCINAP
Supplementary Figure 1g
kDa
26
43
hCINAP
β-actin
Supplementary Figure 2d
kDa
72
43
SENP3
β-actin
Supplementary Figure 2a
kDa
72
55
43
34
26
SENP3
NPM1
hCINAP
Supplementary Figure 2b
kDa
72
43
34
26
SENP3
NPM1 mutant
hCINAP
SENP3
NPM1 mutant
hCINAP
Supplementary Figure 3a
kDa
43
34
26
p-T199 NPM1
β-actin
NPM1
Flag-hCINAP
Supplementary Figure 3c
kDa
43
34
p-T199 NPM1
HA-NPM1 WT/K263R/T199A
β-actin
Supplementary Figure 3e
kDa
43
34
SUMO-NPM1
HA-NPM1 WT/ K263R/T199A
β-actin
Supplementary Figure 3b
kDa
34
43
26
Ubi-NPM1
NPM1
β-actin
Flag-hCINAP
Supplementary Figure 3d
kDa
43
26
p-T199 NPM1
Flag-NPM1 K263R
Flag-hCINAP
hCINAP
Supplementary Figure 3f
kDa
43
26
34
SUMO-NPM1
Flag-hCINAP
NPM1
17
43
γ-H2AX
β-actin
Supplementary Figure 4a
kDa
26
17
Ub-H2A
H2A

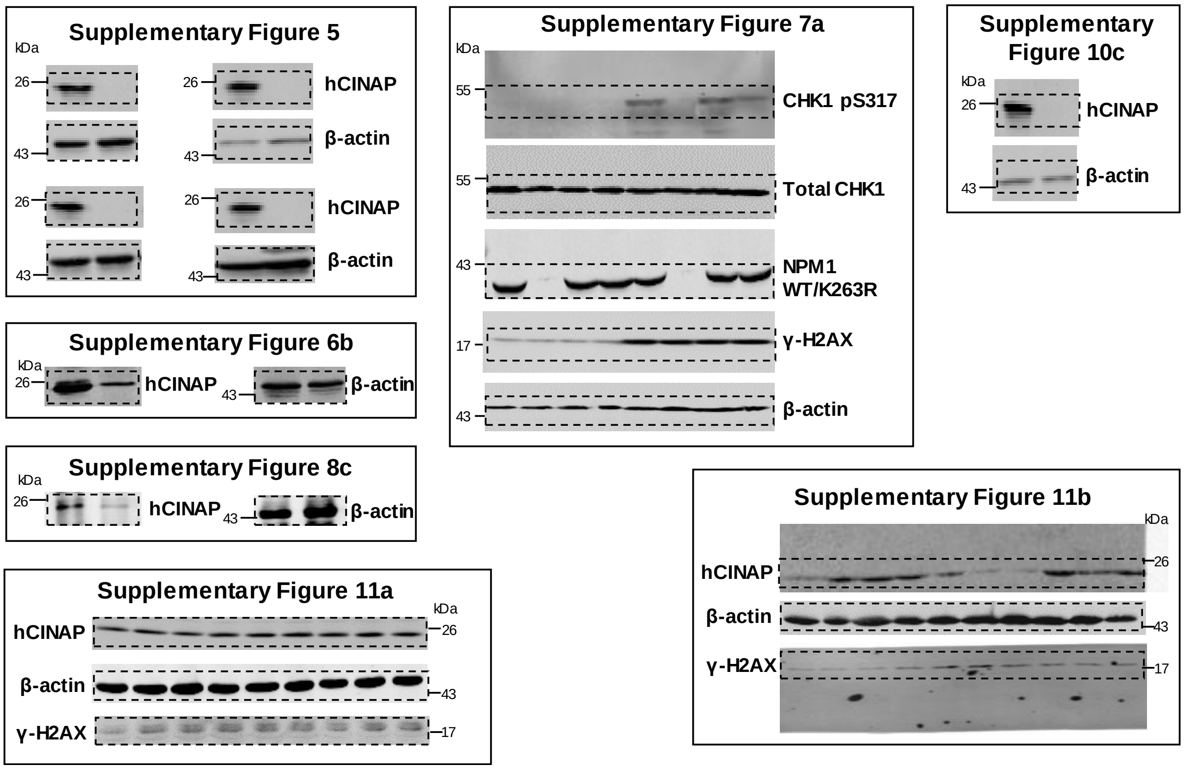

Supplementary Figure 13 | Uncropped immunoblots. Unprocessed images of scanned immunoblots shown in Figures and Supplementary Figures of the manuscript are provided.

Supplementary Methods

shRNAs for knockdown of hCINAP and NPM1

To generate recombinant lentivirus vectors expressing hCINAP-shRNA or non-silencing control-shRNA, hCINAP-shRNA-1: 5'-CAG AGU AGU UGA UGA GUUA-3', hCINAP-shRNA-2: 5'-GAG AGA AGG UGG AGU UAU U-3', and non-silencing control-shRNA: 5'-UUC UCC GAA CGU GUC ACG U-3' were cloned into the pGCSIL-Puromycin lentivirus vector (GeneChem, China). The pLVX-shRNA2 lentivirus vector expressing the NPM1-shRNA oligonucleotides was acquired as a gift from Dr. Jing Yi.

Immunoblot analyses

For Immunoblotting, protein samples were separated via SDS–PAGE followed by transfer to a nitrocellulose filter membrane (PALL). The membrane was first blocked with 5% milk and then sequentially incubated with the indicated primary and secondary antibodies.

Cell apoptosis assay

For the cell apoptosis analysis, cells were harvested by using the Annexin V-FITC Apoptosis Detection Kit (Life Technology). Cells were washed with PBS and stained with Annexin V and propidium iodide. Apoptosis was determined by FACS analysis.

Clonogenic survival assay

Stable cells were seeded at a low density and irradiated with IR at 0, 2, 4, or 8 Gy, or they were treated with 0, 0.1, 0.5, 1 μM DNR; 0, 0.5, 1, 2 μM HD-Ara-C, respectively. The cells were then cultured at 37ºC for at least 14 days. Colonies were stained with 2% methylene blue and 50% ethanol for 10 minutes. The numbers of visible cell clones were directly counted under a microscope.

Co-immunoprecipitation

To detect the role of hCINAP in mediating the interaction between NPM1 and SENP3, HEK293T cells transfected with the indicated plasmids were harvested and subjected to IP assays using the indicated antibodies. Cell lysate immunoprecipitations were performed using the indicated primary antibody and protein A/G agarose beads (Sigma) at 4 °C. The immunocomplexes were then washed with HEPES lysis buffer four times. Both lysates and immunoprecipitates were examined using the indicated primary antibodies followed by detection with the related secondary antibody.

Immunoprecipitation

HEK293T cells were transfected with the Flag-tagged hCINAP or NPM1 plasmids using polyethylenimine (PEI). At 48 hours after transfection, cells were harvested and lysed with NTEN buffer [20 mM Tris-HCl, pH 7.5, 150 mM NaCl, 10% glycerol, 0.5% NP40, 10 mM NaF, 1 mM phenylmethylsulfonyl fluoride (PMSF), 1 μg ml^{-1} leupeptin, 1 μg ml^{-1} aprotinin]. Following ultra-centrifugation at 440,000 g for 15 min at 4°C, the supernatant was incubated with anti-Flag M2-conjugated agarose beads for 4 hours. The beads

were then spun down and washed four times with IP buffer [20 mM Tris-HCl, pH 7.5, 150 mM NaCl, $MgCl_2$ 5 mM, 10% glycerol, 0.1% NP40, 1 mM dithiothreitol (DTT), 1 mM PMSF]. Subsequently, the complexes were eluted with IP buffer containing 400 µg ml^{-1} 3×Flag peptide and analyzed via SDS-PAGE and mass spectrometry.

His-SUMO pull-down analysis

HEK293T cells were transfected with His-SUMO3 with or without Flag-hCINAP. The effect of hCINAP on endogenous NPM1 sumoylation was examined by His-SUMO pull-down analysis.

Protein purification and *in vitro* assays

For *in vitro* pull-down assays, GST-NPM1 was incubated with His-hCINAP or His-RAP80 in PBS buffer at 4°C for 1 hour. The beads were washed with PBS buffer and boiled with 2×SDS loading buffer, followed by immunoblotting.
