# Supplementary material for: hCINAP regulates the DNA-damage response and mediates the resistance of acute myelocytic leukemia cells to therapy
Source: Nat Commun. 2019 Aug 23;10:3812. doi: 10.1038/s41467-019-11795-5 (PMC6707248; doi:10.1038/s41467-019-11795-5)
Supplement: Supplementary file 3 — Reporting Summary [file 41467_2019_11795_MOESM3_ESM.pdf]

nature research

Corresponding author(s): Xiaofeng Zheng

Last updated by author(s): Jul 18, 2019

Reporting Summary

Nature Research wishes to improve the reproducibility of the work that we publish. This form provides structure for consistency and transparency in reporting. For further information on Nature Research policies, see Authors & Referees and the Editorial Policy Checklist .

Statistics

For all statistical analyses, confirm that the following items are present in the figure legend, table legend, main text, or Methods section.

n/a	Confirmed	
☐	☒	The exact sample size (n) for each experimental group/condition, given as a discrete number and unit of measurement
☐	☒	A statement on whether measurements were taken from distinct samples or whether the same sample was measured repeatedly
☐	☒	The statistical test(s) used AND whether they are one- or two-sided<br>*Only common tests should be described solely by name; describe more complex techniques in the Methods section.*
☐	☒	A description of all covariates tested
☐	☒	A description of any assumptions or corrections, such as tests of normality and adjustment for multiple comparisons
☐	☒	A full description of the statistical parameters including central tendency (e.g. means) or other basic estimates (e.g. regression coefficient) AND variation (e.g. standard deviation) or associated estimates of uncertainty (e.g. confidence intervals)
☒	☐	For null hypothesis testing, the test statistic (e.g. F, t, r) with confidence intervals, effect sizes, degrees of freedom and P value noted<br>*Give P values as exact values whenever suitable.*
☒	☐	For Bayesian analysis, information on the choice of priors and Markov chain Monte Carlo settings
☐	☒	For hierarchical and complex designs, identification of the appropriate level for tests and full reporting of outcomes
☐	☒	Estimates of effect sizes (e.g. Cohen's d, Pearson's r), indicating how they were calculated

Our web collection on statistics for biologists contains articles on many of the points above.

Software and code

Policy information about availability of computer code

Data collection	Odyssey; ZEN 2009 Light Edition(Carl Zeiss); NIS (Nikon); FlowJo V10; Case Viewer
Data analysis	GraphPad Prism V7; Image J; Comet Assay IV software (Perceptive Instruments, UK); Imaris 9.0.2. (Bitplane); RStudio; Image-Pro Plus 6.0 ; Volocity

For manuscripts utilizing custom algorithms or software that are central to the research but not yet described in published literature, software must be made available to editors/reviewers. We strongly encourage code deposition in a community repository (e.g. GitHub). See the Nature Research guidelines for submitting code & software for further information.

Data

Policy information about availability of data

All manuscripts must include a data availability statement. This statement should provide the following information, where applicable:

- Accession codes, unique identifiers, or web links for publicly available datasets
- A list of figures that have associated raw data
- A description of any restrictions on data availability

All the data supporting the findings of this study are available from the corresponding author on reasonable request.

Field-specific reporting

Please select the one below that is the best fit for your research. If you are not sure, read the appropriate sections before making your selection.

☒ Life sciences ☐ Behavioural & social sciences ☐ Ecological, evolutionary & environmental sciences

Life sciences study design

All studies must disclose on these points even when the disclosure is negative.

Sample size	For human samples (healthy control and AML patients, 13 samples in each group), no statistical method was used to predetermine sample size due to the availability. For animal study, fig. 8 related experiment used 30 mice with same batch of birth. Unless explicitly stated, 3 independent experiments were performed to achieve Student's t-test analysis.
Data exclusions	No data were excluded for this study.
Replication	The shown experiments could successfully and reliably be replicated and reproduced.
Randomization	Human samples were not randomized. For animal study, all the animals were randomly divided into 2 groups for experiments.
Blinding	No blinding.

Reporting for specific materials, systems and methods

We require information from authors about some types of materials, experimental systems and methods used in many studies. Here, indicate whether each material, system or method listed is relevant to your study. If you are not sure if a list item applies to your research, read the appropriate section before selecting a response.

Materials & experimental systems

n/a	Involved in the study	
☐	☒	Antibodies
☐	☒	Eukaryotic cell lines
☒	☐	Palaeontology
☐	☒	Animals and other organisms
☐	☒	Human research participants
☒	☐	Clinical data

Methods

n/a	Involved in the study	
☒	☐	ChIP-seq
☐	☒	Flow cytometry
☒	☐	MRI-based neuroimaging

Antibodies

Antibodies used	All antibodies are listed in Supplementary.<br>Phospho-Histone H2A.X (Ser139) (05-636, 1:2000 dilution) was from Millopore.<br>Anti-Myc (M047-3), anti-GST (M071-3), anti-β-actin (PM053, 1:2000 dilution), anti-Lamin B1 (PM064), anti-His (D291-3), and anti-α-Tubulin (M175-3) were from Medical & Biological Laboratiries (USA).<br>Monoclonal anti-Flag (M2; F3165) and anti-HA (HA-7; H9658) were from Sigma (USA).<br>Anti-NPM1 (#3542), anti-BRCA1 (#9010; 1:500 dilution) and anti-SENP3 (D20A10) were purchased from Cell Signaling Technology.<br>IRDye 800CW goat anti-mouse (926-32210) and IRDye 800CW goat anti-rabbit (926-32211) were purchased from LI-COR Bioscience (USA). Fluorescein isothiocyanate-conjugated goat anti-mouse IgG (ZF-0312) and TRITC-conjugated anti-rabbit IgG (ZF-0316) were purchased from ZSGB-Bio (China).<br>Rabbit polyclonal anti-hCINAP was generated by immunizing a rabbit with the purified hCINAP protein.
Validation	Unless indicated, all the antibodies were used in 1:1000 dilution. The commercial antibodies were validated based on the information on the manufacturers' instructions. Additional validation was done by the use of shRNA treated samples as negative control.

Eukaryotic cell lines

Policy information about cell lines

Cell line source(s)	KG-1α, OCI-AML2, OCI-AML3, U2OS, HeLa, and 293T cell lines were purchased from ATCC. Mice bone marrow cells and peripheral blood white blood cells were isolated in the lab.
Authentication	Cell lines were examined for their morphology by microscopy and for their protein expression by immunoblot.
Mycoplasma contamination	All of the cells used were regularly tested for mycoplasma contamination. The cell lines were not tested for mycoplasma contamination.
Commonly misidentified lines (See ICLAC register)	No commonly misidentified cell lines were used.

Animals and other organisms

Policy information about studies involving animals; ARRIVE guidelines recommended for reporting animal research

Laboratory animals	All animal studies were conducted in accordance with relevant guidelines and regulations and were approved by the Ethics Committee of Peking University.
Wild animals	n/a
Field-collected samples	n/a
Ethics oversight	The investigation was performed under approval by the Ethics Committee of Peking University.

Note that full information on the approval of the study protocol must also be provided in the manuscript.

Human research participants

Policy information about studies involving human research participants

Population characteristics	The total samples of AMLs included 6 females and 7 males with ages from 24 to 80 years.<br>The total samples of the healthy controls included 8 females and 5 males with ages from 19 to 78 years.
Recruitment	The peripheral blood samples were collected the first time the patients were diagnosed with acute myelocytic leukemia (AML) at the Peking University People's Hospital according to the guidelines of the ethics committees.<br>The blood of healthy patients without any sign of hematological malignancies was collected at Peking University Hospital.
Ethics oversight	Experiments conform to legal and ethics requirements.

Note that full information on the approval of the study protocol must also be provided in the manuscript.

Flow Cytometry

Plots

Confirm that:

- [x] The axis labels state the marker and fluorochrome used (e.g. CD4-FITC).
- [x] The axis scales are clearly visible. Include numbers along axes only for bottom left plot of group (a 'group' is an analysis of identical markers).
- [x] All plots are contour plots with outliers or pseudocolor plots.
- [x] A numerical value for number of cells or percentage (with statistics) is provided.

Methodology

Sample preparation	Detailed of sample preparation is described in Methods.<br>Apoptosis analysis: Apoptotic cells were quantitated using Annexin V and PI staining according to the manufacturer's protocol of Annexin V-FITC Apoptosis Detection Kit (Life Technology). In brief, cells were harvested and washed with PBS and stained with Annexin V and propidium iodide. Apoptosis was determined by FACS (FACSCanto, BD Biosciences).<br>Double strand DNA damage repair efficiency: hCINAP+/+ and hCINAP-/- cells were co-transfected with DR-GFP, an I-SceI expression vector, and a DsRed plasmid. Cells were harvested and washed with 1×PBS 48 hours after transfection. Green (EGFP) and red (DsRed) fluorescence was measured by FACS on a FACSVerse instrument (BD Biosciences, USA). The ratio of EGFP and DsRed double-positive cells to DsRed positive cells was taken as the repair efficiency. The results were normalized to those of hCINAP+/+ cells.
Instrument	Apoptosis was determined by FACS (FACSCanto, BD Biosciences).
Software	BD CellQuest™ Pro Software; FlowJo V10
Cell population abundance	In each experiment group, we randomly selected ten thousand cells from the overall.
Gating strategy	Cells were gated based on forward and side scatter plots, only avoiding debris and aggregates and no extensive gating strategy was used. Especially in Double strand DNA damage repair efficiency, U2OS-DR-GFP cells that were only transfected with DsRed but lacked I-SceI were considered as the negative control (background level of HR) to define the gate.

- [x] Tick this box to confirm that a figure exemplifying the gating strategy is provided in the Supplementary Information.
