# Supplementary material for: hCINAP regulates the DNA-damage response and mediates the resistance of acute myelocytic leukemia cells to therapy
Source: Nat Commun. 2019 Aug 23;10:3812. doi: 10.1038/s41467-019-11795-5 (PMC6707248; doi:10.1038/s41467-019-11795-5)
Supplement: Supplementary file 4 — Description of Additional Supplementary Files [file 41467_2019_11795_MOESM4_ESM.docx]

Description of Additional Supplementary File

**Supplementary Data 1:** Source data of Figure 1h, 2a and 4a.

Spreadsheet 1：Source data of Figure 1h. Expression of hCINAP in AML patients relative to that of healthy controls was analyzed by using the TCGA database.

Spreadsheet 2: Source data of Figure 2a. IP-mass spectrometry dataset of potential hCINAP-interacting proteins.

Spreadsheet 3: Source data of Figure 2a. IP-mass spectrometry dataset of potential hCINAP-interacting proteins under IR treatment.

Spreadsheet 4: Source data of Figure 4a. IP-mass spectrometry dataset of potential NPM1-interacting proteins.

Spreadsheet 5: Source data of Figure 4a. IP-mass spectrometry dataset of potential NPM1-interacting proteins under IR treatment.
