# Supplementary material for: hCINAP regulates the DNA-damage response and mediates the resistance of acute myelocytic leukemia cells to therapy
Source: Nat Commun. 2019 Aug 23;10:3812. doi: 10.1038/s41467-019-11795-5 (PMC6707248; doi:10.1038/s41467-019-11795-5)
Supplement: Supplementary file 2 — Peer Review [file 41467_2019_11795_MOESM2_ESM.pdf]

Reviewers' comments:

Reviewer #1 (Remarks to the Author):

In this manuscript, the authors discovered a novel function of hCINAP and NPM in response to DSBs. They found that upon ionizing irradiation, hCINAP translocates from cytosol to the nucleus, and recruits SENP3 to deSUMOylate NPM1, which in turn promotes BRCA1 recruitment to facilitate pathway choice of DSB repair through homologous recombination. Given that down-regulation of hCINAP, a phenomenon observed in AML patients, results in increased apoptosis, the work also suggests that hCINAP is a potential target for overcoming drug resistance in AML treatment. In general, the manuscript provides evidence supporting a novel mechanism by which hCINAP regulates DNA damage response and DSB repair of pathway choice. The experiments were essentially well done. However, the manuscript can be further improved if the following issues are addressed.

1. The authors showed that BRCA1 interacts with NPM1 interaction by IP, and that NPM1 knockdown inhibits BRCA1 foci formation. They therefore concluded that NPM1 sumoylation promotes accumulation of BRCA1 foci in the presence of DSB. However, they did not show if NPM sumoylation affects the BRCA1-NPM1 interaction directly. The authors can easily determine this by measuring BRCA1 recruitment and its interactions with NPM1 in cells expressing NPM1 WT or NPM1 K263R.

2. RAD51 is an essential component for DSB repair via HR. Does NPM sumoylation promote RAD51 recruitment and RAD51-mediated end resection?

3. The authors showed that hCINAP knockout restores 53BP1 foci formation to promote NHEJ. If this is indeed the case, activation of key components of the NHEJ pathway, e.g., DNA-PKcs phosphorylation, should be detected.

4. Given that hCINAP knockouts delay 53BP1 foci formation and promote NHEJ activity, which induces hypermutation. If this is true, hCINAP-KO cells or NPM1 sumoylation deficient cells and AML patient cells should display a mutator phenotype. Are these true?

5. There are some grammar errors. For example, the sentence “In this study, we found that hCINAP, a new protein dynamically involved in repair of double-stranded DNA breaks, the expression level of which is associated with the prognosis of AML patients” in the Abstract is a phrase.

6. Figure 1c. The letter ‘n’ of Tubulin is not in line with the whole word.The authors have nicely shown the NPM1 domain responsible for the NPM1-hCINAP interaction. Have they determined the hCINAP domain for the interaction? If they have, it would be better to show it here. However, it is not essential to perform the experiment if they don’t have the data.

7. The description of figures S2c and S2d cannot be found in the paper.

8. In figure 3e, the merged fluorescence of NPM1 and hCINAP should also be shown and the time course should be longer to match figure 3f. In some figures, why the band of sumo-NPM1 is located around 34 kDa?

9. In figure 4c, IR treatment seems to be mislabeled. Also, in figure 4d, the authors should explain

why the sumoylation levels of NPM1 are the same in lane 3 and lane 1.
10. In Figure 5c, NPM1 foci and co-localization between NPM1 and BRCA1 should be shown.

11. There is no figure 5d, which appears in the text.

12. The mouse KO data shown in Figures 8a and 8b are pretty weak, and added no additional insights to the paper. In my opinion, the data should be deleted from the manuscript. Instead, the authors confirm their human cell findings in the MEF cell lines, rather than the animals.

Reviewer #2 (Remarks to the Author):

Xu et al reported that hCINAP, a novel DDR protein, got recruited to DSBs and facilitates SENP3 to de-sumoylate NPM1. Xu also showed that sumoylated NPM1 recruited RAP80-BRCA1 complex but inhibited CTIP and RAD51 recruitments. Depleted of hCINAP sustained the NPM1 sumoylation, which in turn inhibited HR and promoted NHEJ. The authors introduced new factors into DDR in this manuscript and the concept is novel. However, the mechanistic links between NPM1 sumoylation and BRCA1 complex recruitment is weak. The major unanswered question is how NPM1 sumoylation regulates the recruitment of BRCA-A complex. Does NPM1 sumoylation directly load BRCA1-A complex or affect RNF168 dependent H2A-Ub? These questions should be addressed in this manuscript.
Major points

1. As mentioned above, although NPM1 interacted with BRCA1, there is no evidence showed that NPM1 sumoylation directly load RAP80-BRCA1. Does NPM1 sumoylation affect H2A-Ub? The author should exam MDC1, RNF8, RNF168 and FK2 IRIF in NPM KR mutant or hCINAP deficient cell lines. If H2A-Ub is not affected, alternative explanation is NPM1 sumoylation directly recruited RAP80-BRCA1. Previous publication (J Biol Chem. 2012 Jul 20; 287(30): 25510–25519.) showed both UB and SUMO modification is coordinated to recruit Rap80 and BRCA1 to DNA damage sites. RAP80 contains a SIM domain, which directly bind SUMO2. It will be interesting to exam whether NPM1 sumoylation directly interacted with the SIM domain of RAP80 in vivo and in vitro. NPM1 WT and KR mutant should be utilized to examine the binding between NPM1 and RAP80 with/without IR. NPM1 sumoylation should be generated totally in vitro and examine its binding with RAP80 SIM domain.

2. The endogenous and exogenous hCINAP localization in cell looks totally different (Fig 1a vs 1d). The author should explain it. endogenous hCINAP should be stained following laser microirradiation treatment.

3. Previous publication (J Biol Chem. 2012 Jul 20; 287(30): 25510–25519.) showed RAP80 bind SUMO2 bit not SUMO1. It will be interesting to examine the SUMO1/2 modification on NPM1.

4. Does SUMO-NPM1 regulate itself recruitment to DSBs? The author should examine the NPM1 recruitment in hCINAP deficient and NPM1 KR mutant background.

5. In Fig 4d, compared SENP3 wt and -/- groups , why SUMO-NPM1 didn’t change?

6. To my understanding, this manuscript demonstrated that hCINAP got recruited following DNA damage and promoted SENP3-depedent desumoylation of NPM1, in turn dissociated RAP80 from DSBs and facilitated CTIP dependent DNA resection and promote HR. The author should rewrite some

sentences in abstract. “After the damaged site is repaired, hCINAP prevents excessive repair by reducing NPM1 sumoylation and promoting disassembly of BRCA1 from damaged sites.”

Minor points
1. In S2d, The label should be FLAG-NPM1 blot should be K263R but not T199A.
2. “Moreover, hCINAP enhanced the interaction between NPM1 and the protease SENP3 both in vitro and in vivo (Fig 4b,c)” is not correct. There is no in vitro experiment performed.

Reviewer #3 (Remarks to the Author):

In the present paper Xu et al. show that an ATPase protein dynamically involved in repair of double-stranded DNA breaks, hCINAP, plays a crucial role in AML. Specifically, when DNA double strand break occurs, hCINAP translocates from the cytosol to the nucleus, preventing excessive repair by reducing NPM1 sumoylation and promoting disassembly of BRCA1 from damaged sites. As a result, decreased hCINAP expression is associated with higher overall-survival rate in AML patients. Overall, Authors provide mechanistic insights into the function of hCINAP during the DNA damage response and correlation with AML suggest its modulation as promising target for anti-AML therapies.

This is a nicely conducted study with interesting results, which are clearly presented and properly interpreted. The bigger concern regards used experimental models which are not in line with the specific focus on AML. Authors performed an elegant set of experiments on U2OS and HEK293T cells achieving innovative results by using co-IP assays and mass spectrometry. Next, hypothesis validation analyses were performed on single AML cell line (KG1α). It is opinion of this referee that a more appropriate in vivo model (primary AML cells) as well as more AML cell lines should be used to validate hCINAP-SENP3-NPM1 link on AML. Remarkably parallel analyses on NPM-1WT and NPM-1mut cells (OCI-AML2 and OCI-AML3, respectively) does worth to be run.
Following are few suggestions that could make present paper more conclusive:

1. Mice used model does not focus on AML and appears not so crucial. Did you plan to use more AML-focused models to confirm data (xenograft or PDX models)?
2. A parallel densitometry graph would make results showed in Figure 1c more conclusive.
3. Did you analyze genomic background (i.e. NPM-1 molecular status) of AML patients showed in Figure 1h?
4. AML hCINAP levels need to be measured after different genotoxic stress including DNA damaging agents (DNR or ARAC).
5. Figure 3D does not show clear difference of Sumo-NPM1 on hCINAPKO vs hCINAPWT following RT exposure. A densitometric analysis would improve these conclusions.
6. In the Abstract section the sentence at 4th line is mis-leading, please proof-edit it.
7. In the discussion section at 24th line a typos occurred (cooperatel)
8. Please check typos mistakes and text spacing throughout the manuscript

Reviewers' comments:

Reviewer #1 (Remarks to the Author):

In this manuscript, the authors discovered a novel function of hCINAP and NPM in response to DSBs. They found that upon ionizing irradiation, hCINAP translocates from cytosol to the nucleus, and recruits SENP3 to deSUMOylate NPM1, which in turn promotes BRCA1 recruitment to facilitate pathway choice of DSB repair through homologous recombination. Given that down-regulation of hCINAP, a phenomenon observed in AML patients, results in increased apoptosis, the work also suggests that hCINAP is a potential target for overcoming drug resistance in AML treatment. In general, the manuscript provides evidence supporting a novel mechanism by which hCINAP regulates DNA damage response and DSB repair of pathway choice. The experiments were essentially well done. However, the manuscript can be further improved if the following issues are addressed.

1. The authors showed that BRCA1 interacts with NPM1 interaction by IP, and that NPM1 knockdown inhibits BRCA1 foci formation. They therefore concluded that NPM1 sumoylation promotes accumulation of BRCA1 foci in the presence of DSB. However, they did not show if NPM sumoylation affects the BRCA1-NPM1 interaction directly. The authors can easily determine this by measuring BRCA1 recruitment and its interactions with NPM1 in cells expressing NPM1 WT or NPM1 K263R.

Response:

Thanks for this valuable suggestion. To investigate the direct link between NPM1 SUMOylation and BRCA1 recruitment, we detected the subcellular localization of BRCA1 and NPM1 WT/K263R using an immunofluorescence assay. IR-induced BRCA1 foci mainly localized in the nucleus, while NPM1 WT and K263R showed different subcellular localization. NPM1 WT localized in both the nucleus and the cytosol, but NPM1 K263R did not show nuclear location anymore (Figure 4d). This observation is consistent with previous studies showing that SUMOylation of NPM1 is required for its nuclear localization (Nakagawa, M. et al., N Engl J Med. 2005; Liu X, et al., Proc Natl Acad Sci U S A. 2007). Thus BRCA1 co-localizes with NPM1 WT but not NPM1 K263R. In addition, we found that under the same IR (6 Gy) treatment, the number of BRCA1 foci in NPM1 K263R cells was fewer than that in NPM1 WT cells (Figure 4d). Moreover, this result was confirmed with a co-IP assay. We found that BRCA1 interacted only with NPM1 WT but not NPM1 K263R (Figure 4e in the revision). A proximity ligation assay (PLA) was also performed, which enabled the detection of protein interaction *in situ* with high specificity and sensitivity. NPM1 WT and BRCA1 interacted mainly in the nucleus (Figure 4f). Taken together, these results indicate that NPM1 SUMOylation not only promotes the recruitment of BRCA1, but also affects NPM1-BRCA1 protein interaction.

We added these new data to the revised manuscript as Figure 4d, e, and f.

2. RAD51 is an essential component for DSB repair via HR. Does NPM sumoylation promote RAD51 recruitment and RAD51-mediated end resection?

Response:

Thank you for the helpful suggestion. Through a co-IP experiment, we found that RAD51 could only bind with NPM1 WT but not NPM1 K263R (Figure 5j). In addition, an immunofluorescence

assay was performed and the results showed that the number of IR-induced RAD51 foci in NPM1 K263R cells was less than that of NPM1 WT cells (Figure 5k). These results indicate that NPM1 SUMOylation promotes RAD51 recruitment.

CHK1 is one of the ATR substrates. CHK1 phosphorylation is critical for RAD51-mediated end resection. We, therefore, examined the effect of NPM1 SUMOylation on CHK1 phosphorylation. We compared the abundance of p-CHK1 and the efficiency of HR in K263R mutant cells with that of NPM1 WT cells. IR treatment promoted the phosphorylation of CHK1, which was inhibited by NPM1 knockdown, and re-expression of NPM1 WT but not K263R mutant could significantly recover p-CHK1 abundance (Supplementary Figure S7a). These data suggest that SUMOylation of NPM1 is important for the phosphorylation of CHK1. Furthermore, measurement of HR pathway repair efficiency showed that NPM1 endogenous expression along with its SUMOylation are necessary for efficient HR repair (Supplementary Figure S7b). These results indicate that NPM1 SUMOylation promotes RAD51 recruitment and RAD51-mediated end resection.

We added the new results to the revised manuscript as Figure 5j, 5k, supplementary Figure S7a, S7b.

3. The authors showed that hCINAP knockout restores 53BP1 foci formation to promote NHEJ. If this is indeed the case, activation of key components of the NHEJ pathway, e.g., DNA-PKcs phosphorylation, should be detected.

Response:

Following the reviewer's suggestion, we assessed the effect of hCINAP on DNA-PKcs phosphorylation by using antibodies targeting DNA damage-induced phosphorylation at Ser-2056 (Wechsler T. et al., *Proc. Natl. Acad. Sci. U.S.A.* 101:1247-1252, 2004). In response to IR treatment, knockout of hCINAP had no effect on the total cellular expression of DNA-PKcs, but increased the abundance of Ser-2056 phosphorylation significantly, while overexpression of hCINAP decreased Ser-2056 phosphorylation (Figure 7d). Moreover, we measured the IR-induced foci formation of DNA-PKcs protein and its Ser-2056 phosphorylated protein in hCINAP-overexpressed cells using an immunofluorescence assay. The results showed that hCINAP overexpression had no influence on DNA-PKcs recruitment, but attenuated the foci formation of phosphorylated DNA-PKcs (Figure 7e, f). These results suggest that hCINAP inhibits key components of the NHEJ pathway including phosphorylated DNA-PKcs.

We added the new results to the revised manuscript as Figure 7d-f.

4. Given that hCINAP knockouts delay 53BP1 foci formation and promote NHEJ activity, which induces hypermutation. If this is true, hCINAP-KO cells or NPM1 sumoylation deficient cells and AML patient cells should display a mutator phenotype. Are these true?

Response:

Thanks for this comment. We also wondered if hCINAP-KO or NPM1 SUMOylation deficiency in AML patient cells displayed a mutator phenotype. To answer this question, we performed experiments to detect the chromosome morphology abnormality and analyzed the mutation frequency of AML patients using the TCGA database as well.

The results of chromosome morphology showed that the peripheral blood (PB) cells from AML patients with a lower abundance of hCINAP accumulated more chromosome breaks and showed

more chromosome instability phenotypes (Supplementary Figure S1f). Meanwhile, the NPM1-depleted AML KG-1α cells that were reintroduced with NPM1 K263R mutant showed more chromosome breaks than that of KG-1α cells rescued with wild-type NPM1 (Supplementary Figure S10b).

When we performing bioinformatics analysis to examine the mutation frequency of AML patients in the TCGA database, no information could be found for NPM1 SUMOylation-deficient AML mutation due to the limited number of samples with available mutation frequency (n=83). We ranked all of the 83 AML patients according to the expression level of hCINAP and NPM1, respectively, defined 'High' or 'Low' expression of the gene as above or below the medium expression value in total AML patients, and performed a one-sided Wilcoxon test. The result showed that lower mRNA levels of both hCINAP and NPM1 potentially correlate with higher mutation frequency (Supplementary Figure S1h, S10a).

5. There are some grammar errors. For example, the sentence "In this study, we found that hCINAP, a new protein dynamically involved in repair of double-stranded DNA breaks, the expression level of which is associated with the prognosis of AML patients" in the Abstract is a phrase.

Response:

Thank you for pointing out the grammar errors. We changed this sentence as "In this study, we identified hCINAP, a novel protein dynamically involved in the repair of double-stranded DNA breaks. The expression level of hCINAP correlates with the prognosis of AML in patients. "

In addition, the entire manuscript has been edited by a native English speaker.

6. Figure 1c. The letter 'n' of Tubulin is not in line with the whole word. The authors have nicely shown the NPM1 domain responsible for the NPM1-hCINAP interaction. Have they determined the hCINAP domain for the interaction? If they have, it would be better to show it here. However, it is not essential to perform the experiment if they don't have the data.

Response:

Thanks for pointing out the mistake, and we corrected it in Figure 1c in the revision.

We also wanted to map the hCINAP domain that interacts with NPM1-hCINAP. We constructed various hCINAP truncations containing the N-terminal nucleoside monophosphate (NMP) kinase domain (1-108 amino acids) and C-terminal non-catalytic domain (109-172 amino acids), respectively, and performed co-IP assays to analyze their interaction with NPM1. However, as the two truncations were too short to express well in eukaryotic cells, we failed to obtain good results.

7. The description of figures S2c and S2d cannot be found in the paper.

Response:

We apologized for the carelessness. We added the description of these figures (Figure S3c and S3d) in the revision.

8. In figure 3e, the merged fluorescence of NPM1 and hCINAP should also be shown and the time course should be longer to match figure 3f. In some figures, why the band of sumo-NPM1 is located around 34 kDa?

Response:

As suggested by the reviewer, we showed the merged fluorescence of NPM1 and hCINAP and listed all the time course in Figure 3f (original Figure 3e) to match the statistical result of Figure 3g (original Figure 3f) in the revision.

The molecular weight of endogenous NPM1 is 38 kDa, and that of its mono-SUMOylated form is 48 kDa. Because we often cut the membrane along the 34 kDa marker ladder, we just labeled the SUMO-NPM1 band above 34 kDa, which was not precise. We remarked the molecular weight in the revision.

9. In figure 4c, IR treatment seems to be mislabeled. Also, in figure 4d, the authors should explain why the sumoylation levels of NPM1 are the same in lane 3 and lane 1.

Response:

Sorry for this mislabeling in the original Figure 4c, as original Figure 4 was merged into Figure 3 in the revision, we corrected it in Figure 3j in the revision.

In the original Figure 4d, we also assumed that the SUMOylation levels of NPM1 in lane 3 should be higher than that of lane 1, as the deSUMOylation enzyme SENP3 is knocked out in lane 3. We repeated this experiment and updated the result of Figure 3k in the revision.

10. In Figure 5c, NPM1 foci and co-localization between NPM1 and BRCA1 should be shown.

Response:

It is difficult to show the NPM1 foci in the original Figure 5c, because the NPM1-depleted cells were infected with lentivirus stably expressing GFP-tagged NPM1 shRNA, and there was no extra channel for assessing NPM1 foci when we performed the immunofluorescent assay (the FITC 488 nm channel is for GFP signals spread in the whole cell, and the TRITC 561 nm channel is used for detection of BRCA1 foci). We thus reconstructed an NPM1 shRNA expression vector with puromycin dihydrochloride, instead of a GFP-tag. In Figure 4d of the revision, we showed both NPM1 and BRCA1 foci, and co-localization between these two proteins

11. There is no figure 5d, which appears in the text.

Response:

We apologize for this mistake; each panel is now described in the text in the revised manuscript.

12. The mouse KO data shown in Figures 8a and 8b are pretty weak, and added no additional insights to the paper. In my opinion, the data should be deleted from the manuscript. Instead, the authors confirm their human cell findings in the MEF cell lines, rather than the animals.

Response:

Thank you for your advice. Following your suggestion, we deleted the data concerning MEF cell survival in the original Figure 8, and moved the results concerning the effect of hCINAP shRNA on KG-1α cell survival to supplementary Figure S8a-c.

Moreover, to demonstrate the function of hCINAP in the regulation of AML, we generated a PDX mice model of AML with depleted hCINAP or wild-type hCINAP (scrambled control). To mimic the clinical medication practice, we administered the “7+3” chemotherapy (HA-Ara-C for seven days and DNR for three days) to the mice and conducted a series of experiments to investigate the effects of hCINAP depletion on cell survival, apoptosis, spleen morphology, and mice survival in response to the chemotherapy. The new results are shown in Figure 8a-g in the

revision. Using PB cells and spleen sections from hCINAP WT and hCINAP-depleted AML mice, we compared the level of SUMOylated NPM1 by IP and PLA assays, respectively (Figure 8j, k, Supplementary Figure S9a), and by PLA assays, we examined the interaction between NPM1 and SENP3 (Figure 8k, Supplementary Figure S9a). We also examined the correlation between BRCA1 foci and NPM1 SUMOylation as shown in Figure 8l and Supplementary Figure S9b.

Reviewer #2 (Remarks to the Author):

Xu et al reported that hCINAP, a novel DDR protein, got recruited to DSBs and facilitates SENP3 to de-sumoylate NPM1. Xu also showed that sumoylated NPM1 recruited RAP80-BRCA1 complex but inhibited CTIP and RAD51 recruitments. Depleted of hCINAP sustained the NPM1 sumoylation, which in turn inhibited HR and promoted NHEJ. The authors introduced new factors into DDR in this manuscript and the concept is novel. However, the mechanistic links between NPM1 sumoylation and BRCA1 complex recruitment is weak. The major unanswered question is how NPM1 sumoylation regulates the recruitment of BRCA-A complex. Does NPM1 sumoylation directly load BRCA1-A complex or affect RNF168 dependent H2A-Ub? These questions should be addressed in this manuscript.

Major points

1. As mentioned above, although NPM1 interacted with BRCA1, there is no evidence showed that NPM1 sumoylation directly load RAP80-BRCA1. Does NPM1 sumoylation affect H2A-Ub? The author should exam MDC1, RNF8, RNF168 and FK2 IRIF in NPM KR mutant or hCINAP deficient cell lines. If H2A-Ub is not affected, alternative explanation is NPM1 sumoylation directly recruited RAP80-BRCA1. Previous publication (J Biol Chem. 2012 Jul 20; 287(30): 25510–25519.) showed both UB and SUMO modification is coordinated to recruit Rap80 and BRCA1 to DNA damage sites. RAP80 contains a SIM domain, which directly bind SUMO2. It will be interesting to exam whether NPM1 sumoylation directly interacted with the SIM domain of RAP80 in vivo and in vitro. NPM1 WT and KR mutant should be utilized to examine the binding between NPM1 and RAP80 with/without IR. NPM1 sumoylation should be generated totally in vitro and examine its binding with RAP80 SIM domain.

Response:

Thank you for your helpful comments. Following your suggestions, we performed several experiments to study the effect of NPM1 SUMOylation on the recruitment of RAP80 and the underlying mechanism.

We first investigated the influence of NPM1 SUMOylation on H2A ubiquitination. Using a His pull-down assay, no difference in H2A ubiquitination was observed in cells expressing Flag-tagged wild-type NPM1 or NPM1 K263R with or without IR treatment (Supplementary Figure S4a). We also assessed the effects of NPM1 SUMOylation or hCINAP on the IR-induced foci formations of MDC1, RNF8, RNF168, and FK2 by immunofluorescence. No significant changes in the foci formation of MDC1, RNF8, RNF168, or FK2 were observed in the NPM K263R mutant or hCINAP-deficient cell lines (Supplementary Figure S4b-e and Supplementary Figure S5a-d). These data indicate that NPM1 SUMOylation does not influence H2A ubiquitination.

We next investigated if NPM1 SUMOylation recruited RAP80-BRCA1. As both UB and SUMO modification coordinate to recruit RAP80 and BRCA1 to DNA damage sites (J. Biol.

Chem. 2012; 287: 25510–25519), we constructed two expression vectors, namely RAP80 full length (FL) and SIM domain-deleted RAP80 (ΔSIM), and examined whether SUMOylated NPM1 directly interacted with the SIM domain of RAP80 *in vivo* and *in vitro*. In cells with depleted NPM1, we first re-expressed NPM1 WT or NPM1 K263R mutant, treated with or without IR, and then performed a co-IP assay to examine if mutation of NPM1 SUMOylation site affects its binding to RAP80. As shown in Figure 6a, IR treatment enhanced the interaction between wild-type NPM1 and RAP80, while the K263R mutant showed no interaction with RAP80. In line with this result, PLA analysis also revealed more interacting PLA signals in cells with NPM1 WT and RAP80 than in the cells with NPM1 K263R-RAP80 (Figure 6b, c). Moreover, we assessed the interaction between NPM1 and RAP80 FL or RAP80ΔSIM. Deletion of the SIM domain abrogated the interaction between NPM1 and RAP80 with/without IR (Figure 6d).

We also confirmed the interaction between SUMOylated NPM1 and the SIM domain of RAP80 by *in vitro* assay. We purified GST-NPM1, His-RAP80 FL, and His-RAP80 ΔSIM proteins, and obtained GST-NPM1-SUMO2 using an *in vitro* SUMOylation Assay Kit (Abcam, ab139470). Then we carried out GST pull-down assays to investigate the effects of NPM1 SUMOylation and SIM deletion on the NPM1-RAP80 interaction. Consistent with the in vivo results, GST-NPM1-SUMO could bind directly to His-RAP80 FL but not His-RAP80 ΔSIM, and no interaction was observed between unSUMOylated GST-NPM1 and His-RAP80 FL or His-RAP80 ΔSIM (Figure 6e). Taken together, we conclude that SUMOylated NPM1 directly interacts with the SIM domain of RAP80 in vivo and in vitro (schematic diagram shown in Figure 6f). These results indicate that the SIM domain of RAP80 is required for its interaction with NPM1 and NPM1 SUMOylation is essential for recruitment of RAP80.to the revised manuscript as Figure 6, Supplementary Figure S4, and Supplementary Figure S5.

2. The endogenous and exogenous hCINAP localization in cell looks totally different (Fig 1a vs 1d). The author should explain it. endogenous hCINAP should be stained following laser microirradiation treatment.

Response:

Thank you for the comments. We also observed the difference between endogenous and exogenous hCINAP localization in our previous work (Ji Y et al., Nat Comm. 2017 May 18). Endogenous hCINAP localizes mainly in the cytoplasm, and could also be observed in the nucleus, while overexpression of GFP-tagged hCINAP resulted in more nuclear localization.

In this study, we performed immunofluorescence and a subcellular fraction assay to examine endogenous hCINAP localization. We found that without DNA damage, the majority of hCINAP remained in the cytosol, while the minority localized to the nucleus. However, in response to DNA damage, hCINAP translocated from the cytoplasm into the nucleus (Figure 1a, 1c). As these phenomena were observed without exogenous hCINAP, we believed that they reflect the actual biological process. When GFP-tagged hCINAP was overexpressed in cells, the nuclear localization of hCINAP appeared stronger. In the meantime, other GFP-tagged proteins, including NPM1 (Figure 3f) and RPL6 (J. Biol. Chem. 2019.294(8):2827–2838), were mainly detected in the nucleus. It is likely that the GFP tag leads to changes in the subcellular localization of exogenous proteins to some extent.

As suggested by the reviewer, we stained endogenous hCINAP following laser micro-irradiation treatment, and added the results as Supplementary Figure S1a in the revision.

Taken together, we believe that despite exogenous GFP-hCINAP showing stronger nuclear localization, the conclusion drawn from Figure 1 is reliable.

3. Previous publication (J Biol Chem. 2012 Jul 20; 287(30): 25510–25519.) showed RAP80 bind SUMO2 bit not SUMO1. It will be interesting to examine the SUMO1/2 modification on NPM1.

Response:

Following this suggestion, we screened the SUMOylation type of NPM1 using His-SUMO pull-down assays and found that NPM1 has all the three types of SUMOylation (SUMO1, SUMO2, and SUMO3) (Figure shown below). However, the only known SUMO-specific protease for NPM1 is SENP3, which specifically cleaves SUMO2 and SUMO3 from its substrate. Thus, we did not examine the SUMO1 modification in our research.

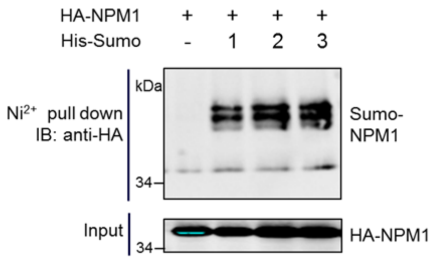

4. Does SUMO-NPM1 regulate itself recruitment to DSBs? The author should examine the NPM1 recruitment in hCINAP deficient and NPM1 KR mutant background.

Response:

Thank you for the helpful suggestion. To find out whether SUMO-NPM1 regulates its own recruitment, we performed micro-irradiation experiments in cells with deficient hCINAP or NPM1 K263R mutant. Additionally, as the reviewer kindly suggested in question 2, we also detected the recruitment of endogenous NPM1 to DSBs. We found that in both hCINAP-deficient and NPM1 K263R mutant cells, NPM1 was recruited to the damage site (Supplementary Figure S10c, S10d), suggesting that SUMOylation of NPM1 does not affect its recruitment to the DSBs, and depletion of hCINAP has no effect as well.

5. In Fig 4d, compared SENP3 wt and -/- groups, why SUMO-NPM1 didn’t change?

Response:

We also assumed that the SUMOylation levels of NPM1 in lane 3 should be higher than that of lane 1, as the deSUMOylation enzyme SENP3 is knocked out in lane 3. We repeated this experiment and updated the result of Figure 3k (as original Figure 4 was merged into Figure 3) in the revision

6. To my understanding, this manuscript demonstrated that hCINAP got recruited following DNA damage and promoted SENP3-depedent desumoylation of NPM1, in turn dissociated RAP80 from DSBs and facilitated CTIP dependent DNA resection and promote HR. The author should rewrite some sentences in abstract. “After the damaged site is repaired, hCINAP prevents excessive repair by reducing NPM1 sumoylation and promoting disassembly of BRCA1 from damaged sites.”

Response:

Following your suggestion, we rewrote the sentence as follows: " Following DSB stimuli,

hCINAP is recruited to damage sites where it promotes SENP3-dependent deSUMOylation of NPM1, which in turn dissociates RAP80 from the damage site and facilitates CTIP-dependent DNA resection and homologous recombination. "

Minor points

1. In S2d, The label should be FLAG-NPM1 blot should be K263R but not T199A.

Response:

Thank you for pointing out the mistake. We corrected the label in Supplementary Figure S3d (original Figure S2d) in the revised version.

2. “Moreover, hCINAP enhanced the interaction between NPM1 and the protease SENP3 both in vitro and in vivo (Fig 4b,c)” is not correct. There is no in vitro experiment performed.

Response:

Thank you for pointing out the mistake. We mistakenly used the word in vitro to express that exogenous NPM1 and exogenous SENP3 also showed stronger binding, regulated by hCINAP; yet it is true that all experiments in Figure 3i, j (original Figure 4b and 4c) are in vivo. We have corrected the wording in the revision.

Reviewer #3 (Remarks to the Author):

In the present paper Xu et al. show that an ATPase protein dynamically involved in repair of double-stranded DNA breaks, hCINAP, plays a crucial role in AML. Specifically, when DNA double strand break occurs, hCINAP translocates from the cytosol to the nucleus, preventing excessive repair by reducing NPM1 sumoylation and promoting disassembly of BRCA1 from damaged sites. As a result, decreased hCINAP expression is associated with higher overall-survival rate in AML patients. Overall, Authors provide mechanistic insights into the function of hCINAP during the DNA damage response and correlation with AML suggest its modulation as promising target for anti-AML therapies.

This is a nicely conducted study with interesting results, which are clearly presented and properly interpreted. The bigger concern regards used experimental models which are not in line with the specific focus on AML. Authors performed an elegant set of experiments on U2OS and HEK293T cells achieving innovative results by using co-IP assays and mass spectrometry. Next, hypothesis validation analyses were performed on single AML cell line (KG1α). It is opinion of this referee that a more appropriate in vivo model (primary AML cells) as well as more AML cell lines should be used to validate hCINAP-SENP3-NPM1 link on AML. Remarkably parallel analyses on NPM-1WT and NPM-1mut cells (OCI-AML2 and OCI-AML3, respectively) does worth to be run. Following are few suggestions that could make present paper more conclusive:

We truly appreciate your evaluation of our study. Regarding your concerns of the experimental models, we performed experiments in multiple AML cell lines including KG-1α, OCI-AML2 (NPM1 WT), OCI-AML3 (NPM1 mutant), AML patients PB cells, and a patient-derived xenograft (PDX) AML-mice model.

We confirmed the interaction between endogenous hCINAP-SENP3 and hCINAP-NPM1 in the OCI-AML2 cell line (Supplementary Figure S2a) and the interaction between endogenous hCINAP-SENP3 and hCINAP-NPM1 mutant in the OCI-AML3 cell line (Supplementary Figure S2b).

In addition, we examined the effect of hCINAP depletion on IR-induced BRCA1 foci in AML peripheral blood (PB) cells and AML mice spleen cells using IF assay (Supplementary Figure S6, S9b).

The metaphase spread experiments were performed using both AML patient PB cells (Supplementary Figure S1f) and the KG-1α cell line (Supplementary Figure S10b), and the chemotherapy and radiotherapy sensitivity assays were carried out in KG-1α cells (Supplementary Figure S8a-c).

In addition, detection of the hCINAP protein expression level, IR-induced apoptosis ratio, and neutral comet assay were performed in both the AML patient PB cells (Figure 1i, 8h, Supplementary Figure S1e, S11) and KG-1α cell line (Supplementary Figure S1g).

Studies using the PDX AML mice model are shown in Figure 8 and described below.

1. Mice used model does not focus on AML and appears not so crucial. Did you plan to use more AML-focused models to confirm data (xenograft or PDX models)?

Response:

Thank you for your valuable suggestion. We finished construction of the AML-focused mice model and carried out the experiments that were not available in our previously submitted manuscript. The new results were added to Figure 8a-g, j-l in the revision. As hCINAP heterozygous mice model and MEF cell survival experiments were not closely related to AML, we deleted the original data in Figure 8a-c, and moved the results concerning the effect of hCINAP shRNA on KG-1α cell survival into Supplementary Figure S8a-c in the revision.

To establish the PDX AML mice model, leukocytes were isolated from the peripheral blood (PB) of an AML patient by gradient centrifugation, and then injected into the tail veins of NOD/SCID donor mice to induce AML. Twenty days later, the donor mice with successfully induced AML disease were sacrificed, the muscles and residue tissues surrounding the femur were removed, and the bone marrow (BM) cells were isolated. Through in vitro proliferation and induction, large amounts of AML-bone marrow hematopoietic stem cells were obtained, which were further infected with lentiviruses expressing GFP-tagged wild-type hCINAP- or knocked down hCINAP. These two types of AML-bone marrow hematopoietic stem cells were then injected into 28 recipient NOD/SCID mice (14 in each group) and acquired AML mice with depleted hCINAP or wild-type hCINAP (scrambled control) cells. The injection day was named as D0.

To mimic the clinical medication practice, we applied the “7+3” chemotherapy (HA-Ara-C for seven days and DNR for three days) to the mice. The beginning of the chemotherapy day was named D10. DNR was administered for three days (D10-D12) plus HA-Ara-C for seven days (D10-D16). We then observed the morphology, movement, and hair loss status of mice, and assessed biochemical criterion and survival rate of the two groups of AML mice. Peripheral blood was collected for TUNEL fluorescence assay to detect cell death, and spleen samples were collected for immunohistochemistry to investigate the expression of hCINAP, NPM1, γ-H2AX, and Ki67. Femur bone marrow was also isolated and subjected to fluorescence-activated cell sorting (FACS) to analyze the proliferation of xenografted GFP tagged bone marrow cells.

The conventional therapy for AML disease is to kill all the white blood cells, and then re-establish the blood circulation system by hematopoietic stem cell transplantation (HSCT). In line with this idea, high levels of drug sensitivity and genomic instability are associated with improved prognosis. Results of the experiments above show that hCINAP-depleted AML cells have higher sensitivity to chemotherapeutics, increased rates of DNA damage and cell death, and slower progression (Figure 8a-g). Decreased hCINAP expression favors complete remission (CR) and subsequent hematopoietic stem cell transplantation (HSCT); it is also associated with a longer lifespan and a reduced relapse, which is in accord with the bioinformatics analysis in Figure 8i.

Using PB cells and spleen sections from hCINAP WT and hCINAP-depleted AML mice, we compared the level of SUMOylated NPM1 (Figure 8j, k, Supplementary Figure S9a), and by PLA assay, we examined the interaction between NPM1 and SENP3 (Figure 8k, Supplementary Figure S9a). We also examined the correlation between BRCA1 foci and NPM1 SUMOylation as shown in Figure 8l and Supplementary Figure S9b.

2. A parallel densitometry graph would make results showed in Figure 1c more conclusive.

Response:

Following the reviewer's suggestion, we performed three independent experiments for Figure 1c, and quantified blots from these three independent experiments using ImageJ, normalizing the intensity of the bands to the untreated lanes (nuclear: lane 1, cytosol: lane 5). The densitometry graph was added in Figure 1c in the revised version. These results indicate that the subcellular localization of hCINAP changed dynamically in the process of DDR.

3. Did you analyze genomic background (i.e. NPM-1 molecular status) of AML patients showed in Figure 1h?

Response:

In the Methods section (on page 10, 1st paragraph), to explain the data collecting methodology for Figure 1h, we wrote "We compiled the datasets of TCGA LAML cancer and GTEx Bone Marrow tissue for differential expression and survival analyses. The sample set included 243 samples (including survival information of 173 patients and 70 normal samples)".

During the data mining process, only the mRNA data of AML patients were available in the TCGA database, but not the mRNA data of healthy people as the control group. To solve this problem, we acquired bone marrow mRNA data of healthy people (normal) from the GTEx Bone Marrow database. We normalized the expression data above and performed the statistical analysis between healthy people and AML patients, shown in Figure 1h. In addition to the bioinformatics result that hCINAP expression is significantly lower in AML patients than healthy people, we also performed western blot analysis from patient samples. Since we were not able to acquire the bone marrow tissue from AML patients in the hospital, we collected the peripheral blood samples of AML patients instead. We then isolated leukocytes from the blood and perform immunoblotting to test hCINAP expression. As shown in Figure 1i and1j, hCINAP expression in AML patients is significantly lower than that of healthy people, confirming the data from the bioinformatics analysis.

While trying to analyze the genomic background of AML patients, we could only look into the SNV of AML patients alone, by calling SNV data with MuSE, MuTect2, SomaticSniper, and VarScan2 function. Yet we were not able to perform a statistical analysis between AML patients

and healthy people, as we couldn't acquire the SNV data of the bone marrow tissue samples in the GTEx database. We find your question particularly intriguing, so we made up for the inadequacy of statistical analysis in the case above with chromosomal assays of AML patients and normal people. We again isolated leukocytes from the peripheral blood of the patients and control group, and observed more morphological deficiencies in the chromosomes of leukocytes from AML patients, as shown in Supplementary Figure S1f. We hope that the results above help answer the question regarding the genomic background.

The number of NPM1 mutations in the LAML cohort range from 5 to 28, according to the 3 calling methods above. The mutation data in tumor samples are based on the SomaticSniper, MuSE, and VarScan2 (5 for SomaticSniper, 8 for MuSE and 28 for VarScan2; No available NPM1 SNV data in MuTect2 results) (figure shown below). Only the samples with at least one mutation in the NPM1 gene are marked as dots in the plots. While we cannot acquire the same data in the control group, it is believed that the mutation number per gene of a healthy sample is close to 0. Additionally, previous studies and review articles also reported that the high mutation rate of NPM1 is a common phenomenon of AML patients; approximately 30% of AML patients have an NPM1 mutation (Vallapureddy R et al., Am J Hematol. 2017 Oct; Nakagawa M et al., N Engl J Med. 2005 Apr 28).

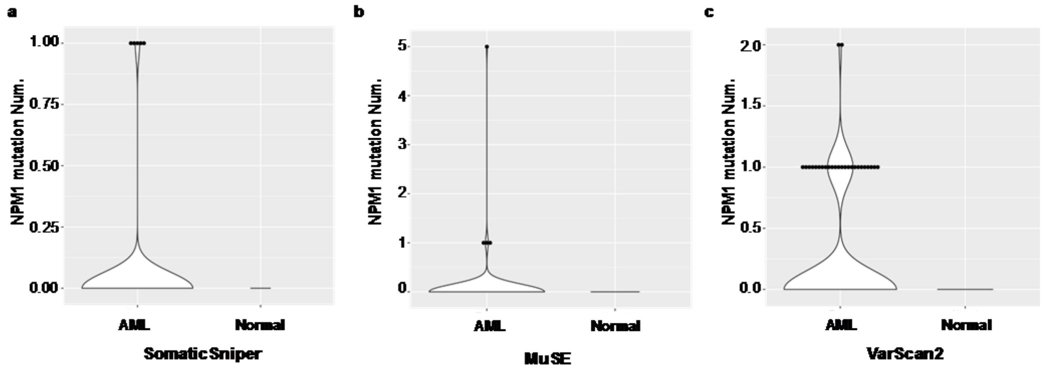

NPM1 mutation analysis in AML patients

4. AML hCINAP levels need to be measured after different genotoxic stress including DNA damaging agents (DNR or ARAC).

Response:

Thank you for your suggestion. To address this comment, leukocytes were isolated from peripheral blood of AML patients and healthy people (as a control group) respectively, and treated with different concentrations of DNR or Ara-C for 24 hours, following the procedure of a related research paper (Bossis, G. et al., Cell Reports, 2014, 1815-1823). The abundance of hCINAP was examined. An increased hCINAP level was observed in AML patients but not healthy people after drug treatment, yet the increased hCINAP expression was still well below that of the healthy people (Supplementary Figure S11).

As hCINAP is important for maintaining genome integrity, together with the result from Figure 8h, we speculated that in response to DNA damage stimuli such as DNR or Ara-C treatment, AML cells with a low abundance of hCINAP are more sensitive to the drug treatment and thus undergo apoptosis. On the other hand, cells might increase chemotherapy resistance by promoting hCINAP expression. Therefore, inhibition of the abundance of

hCINAP together with chemotherapy would increase the therapeutic effect.

5. Figure 3D does not show clear difference of SUMO-NPM1 on hCINAPKO vs hCINAPWT following RT exposure. A densitometric analysis would improve these conclusions.

Response:

Following your suggestion, we performed His pull-down assays at least three times to test the level of NPM1 SUMOylation in hCINAP WT and KO cells at different time points followed IR treatment, and did a densitometric analysis (Figure 3e) to improve the conclusions in Figure 3d. To summarize, the NPM1 SUMOylation reached a peak at 1 hour after IR treatment, which was approximately the same in hCINAP WT and KO cells (Figure 3d, e). Nevertheless, differences were observed at 8 hours after IR treatment, where SUMOylation of NPM1 was much lower in hCINAP WT cells compared to that of KO cells, suggesting that hCINAP functions in the late stage of DNA damage repair as a regulatory component by modulating the level of NPM1 SUMOylation to prevent excessive repair.

We added the new densitometric analysis result in the revision as Figure 3e.

6. In the Abstract section the sentence at 4th line is mis-leading, please proof-edit it.

Response:

Thank you for this helpful comment. We changed this sentence as "In this study, we identified hCINAP, a novel protein dynamically involved in the repair of double-stranded DNA breaks. The expression level of hCINAP correlates with the prognosis of AML in patients."

7. In the discussion section at 24th line a typos occurred (cooperatel)

Response:

Thank you for pointing out the mistake. We corrected this typo in this revision.

8. Please check typos mistakes and text spacing throughout the manuscript

Response:

Following the suggestion from the reviewer, we corrected grammatical mistakes and typos. The manuscript was revised by a professional editor whose native language is English.

REVIEWERS' COMMENTS:

Reviewer #1 (Remarks to the Author):

In this manuscript, the authors discovered a novel function of hCINAP and NPM in response to DSBs. They found that upon ionizing irradiation, hCINAP translocates from cytosol to the nucleus, and recruits SENP3 to deSUMOylate NPM1, which in turn promotes BRCA1 recruitment to facilitate pathway choice of DSB repair through homologous recombination. Given that down-regulation of hCINAP, a phenomenon observed in AML patients, results in increased apoptosis, the work also suggests that hCINAP is a potential target for overcoming drug resistance in AML treatment. In my opinion, the work provides convicing evidence supporting a novel mechanism by which hCINAP regulates DNA damage response and DSB repair of pathway choice. The experiments were well done. The authors did an excellent job in revision. All my questions have been well addressed. I only have a couple of minor concerns.
1. In Figure 5j, the IgG control shows an non-specific RAD51 band, which should be addressed.
2. In my previous comments, there was an inappropriate phrase of "RAD51-mediated end resection". What I wanted to know is whether or not NPM sumoylation regulates RAD51 recruitment and RAD51-mediated DSB repair. The new data provided have answered my question nicely. Thus, please modify the phrase in the manuscript text accordingly, which appears in p19 line 4, p19 line 14, p40 line 11.

Reviewer #2 (Remarks to the Author):

The authors addressed all my questions. The manuscript is well organized. I suggest to accept this manuscript to publish in Nature Communications.

Reviewer #3 (Remarks to the Author):

Authors have addressed all my criticisms and paper is now ready to be published

REVIEWERS' COMMENTS:

Reviewer #1 (Remarks to the Author):

In this manuscript, the authors discovered a novel function of hCINAP and NPM in response to DSBs. They found that upon ionizing irradiation, hCINAP translocates from cytosol to the nucleus, and recruits SENP3 to deSUMOylate NPM1, which in turn promotes BRCA1 recruitment to facilitate pathway choice of DSB repair through homologous recombination. Given that down-regulation of hCINAP, a phenomenon observed in AML patients, results in increased apoptosis, the work also suggests that hCINAP is a potential target for overcoming drug resistance in AML treatment. In my opinion, the work provides convicing evidence supporting a novel mechanism by which hCINAP regulates DNA damage response and DSB repair of pathway choice. The experiments were well done.

The authors did an excellent job in revision. All my questions have been well addressed. I only have a couple of minor concerns.

1. In Figure 5j, the IgG control shows an non-specific RAD51 band, which should be addressed.

Response:

As suggested, the non-specific band in Figure 5j is addressed in this revision.

2. In my previous comments, there was an inappropriate phrase of "RAD51-mediated end resection". What I wanted to know is whether or not NPM sumoylation regulates RAD51 recruitment and RAD51-mediated DSB repair. The new data provided have answered my question nicely. Thus, please modify the phrase in the manuscript text accordingly, which appears in p19 line 4, p19 line 14, p40 line 11.

Response:

Thanks for the corrections. We modified the phrases in the manuscript accordingly.

Reviewer #2 (Remarks to the Author):

The authors addressed all my questions. The manuscript is well organized. I suggest to accept this manuscript to publish in Nature Communications.

Reviewer #3 (Remarks to the Author):

Authors have addressed all my criticisms and paper is now ready to be publishedTITLE PAGE
